# Optimizing the thermoelectric performance of the earth-abundant layered phosphide BaCuP through defect engineering

**DOI:** 10.1039/d6ta05718g

**Published:** 2026-08-28

**Authors:** Romain Claes, Peter P. Russell, Alexander G. Squires, David O. Scanlon

**Affiliations:** a School of Chemistry, University of Birmingham Edgbaston Birmingham B15 2 TT UK r.claes@bham.ac.uk d.o.scanlon@bham.ac.uk

## Abstract

Thermoelectric materials offer a direct route for converting waste heat into electricity, but the discovery of efficient, sustainable, and non-toxic compounds remains a major challenge. Here, we investigate the thermoelectric potential of the layered 1-1-1 phosphide BaCuP using a combination of hybrid density functional theory, defect calculations, Boltzmann carrier transport theory, and machine-learning assisted lattice thermal conductivity simulations. The electronic structure reveals a small indirect band gap of 0.19 eV and strongly anisotropic valence-band dispersion, arising from Cu–P derived states, which favours in-plane hole transport. Defect calculations show that BaCuP is intrinsically strongly p-type due to the very low formation energy of copper vacancies under Cu-poor conditions, yielding high hole concentrations. We further assess K and La doping as routes to tune the carrier concentration, and find that La doping is particularly promising because it slightly reduces the intrinsic hole concentration toward the optimum range for thermoelectric performance. To accurately describe phonon transport, we train a machine-learning interatomic potential and use homogeneous non-equilibrium molecular dynamics, which captures higher-order anharmonic effects beyond conventional third-order approaches. BaCuP is predicted to exhibit a lower lattice thermal conductivity relative to CaCuP due to the heavier Ba. Combining the electronic and thermal transport properties, we predict a peak directionally averaged figure of merit *zT* of about 0.69 at 800 K in La-doped BaCuP, with in-plane values approaching 1 but hindered by the out-of-plane direction. We further show that nanostructuring does not improve the figure of merit in this material, because reducing the mean free path suppresses the carrier transport more strongly than the lattice thermal conductivity. BaCuP nonetheless stands out as a promising phosphide thermoelectric.

## Abstract 2

Defect engineering of the earth-abundant layered phosphide BaCuP: La doping tunes the intrinsic hole concentration towards the optimum, giving a peak *zT* of 0.69 at 800 K, the highest among 1-1-1 phosphides.

## Graphical abstract

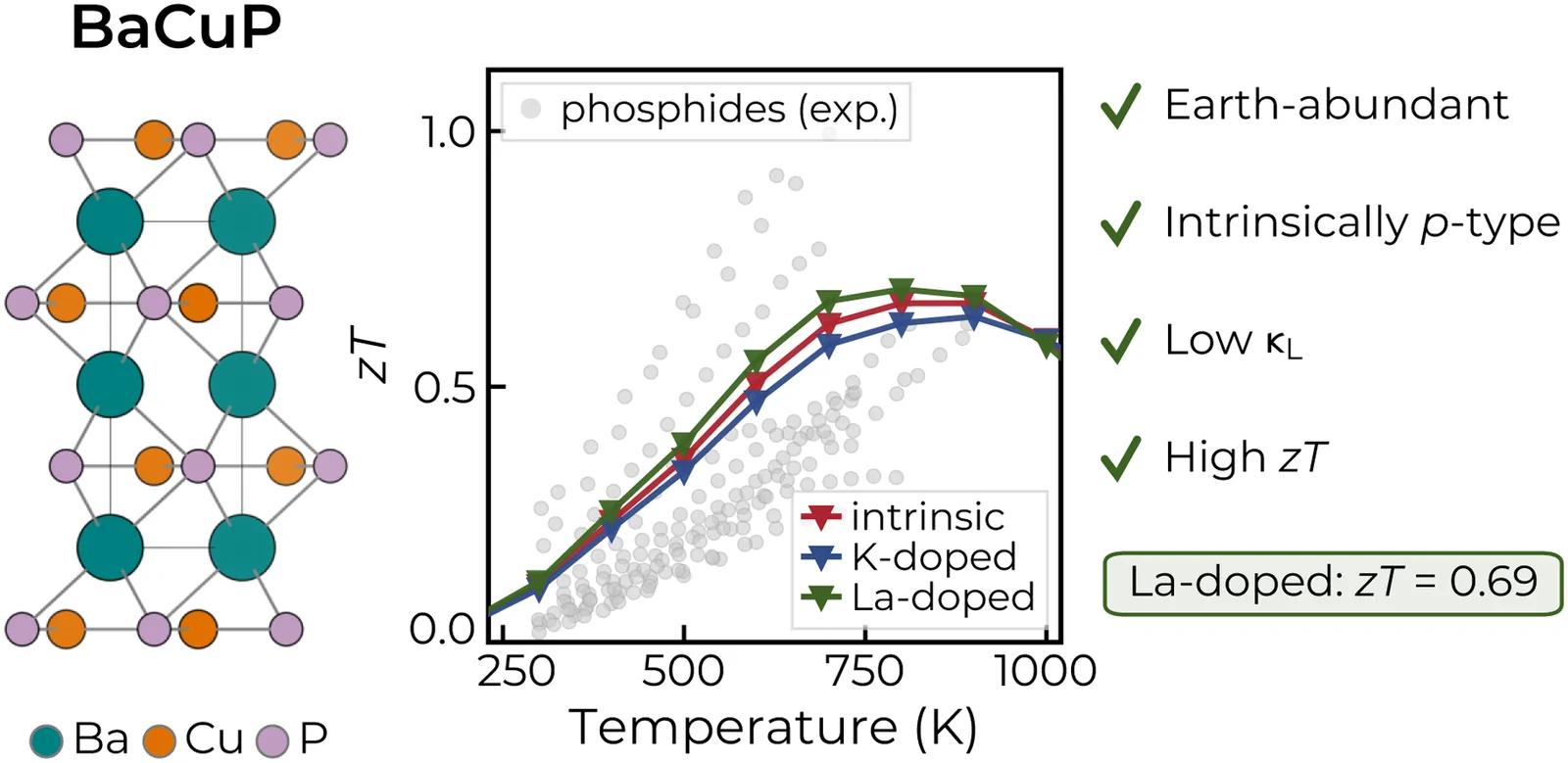

## 1. Introduction

Thermoelectric materials enable the direct conversion of heat into electricity, offering a potential route for both waste-heat recovery and reliable electricity generation.^1–3^ This is particularly attractive as a substantial fraction of the world's energy is ultimately dissipated as waste heat in industrial processes, transportation, or power generation.^4^ Harnessing even a small portion of this lost energy would provide meaningful gains in overall energy efficiency and contribute to emissions reduction. Thermoelectric performance is commonly quantified by the dimensionless figure of merit, *zT*, defined as1
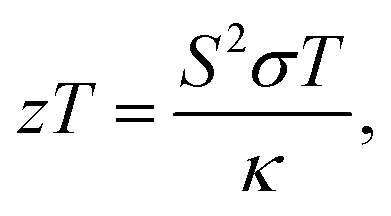
where *S* is the Seebeck coefficient, *σ* the electrical conductivity, *T* the absolute temperature, and *κ* the total thermal conductivity, including the lattice (*κ*_l_) and electronic (*κ*_e_) contributions.^1^ Because the conversion efficiency of thermoelectric devices increases with *zT*, achieving large *zT* values, ideally over a broad temperature window, is key to improving device-level performance.

Despite significant progress, there remains a strong need for thermoelectric materials that combine high performance with low cost and long-term sustainability. In particular, moving beyond state-of-the-art chalcogenide systems such as PbTe,^5,6^ Bi_2_Te_3_,^7,8^ and SnSe^9,10^ motivates the development of alternative compounds composed of earth-abundant, non-toxic elements and amenable to scalable synthesis routes, while maintaining competitive efficiency across relevant operating temperatures. In this context, several classes of materials have been explored, including Sb-based Zintl phases^11–13^ and layered mixed-anion oxides,^14–16^ which have demonstrated promising thermoelectric properties. Metal phosphides represent another emerging class of materials with significant potential for thermoelectric applications, yet they remain comparatively underexplored.^17,18^ Most reported metal phosphides exhibit intrinsically p-type transport behaviour, commonly attributed to small cation deficiencies (*e.g.*, Cu or Ag vacancies) in their crystal structures,^19,20^ with only a limited number of exceptions showing n-type conduction.^21,22^ To date, the highest thermoelectric performance among phosphide-based systems has been achieved in the complex compound Ag_6_Ge_10_P_12_ co-doped with Cu and Ga which yields a maximum *zT* slightly exceeding 1 at 723 K.^23^

**Fig. 1:**
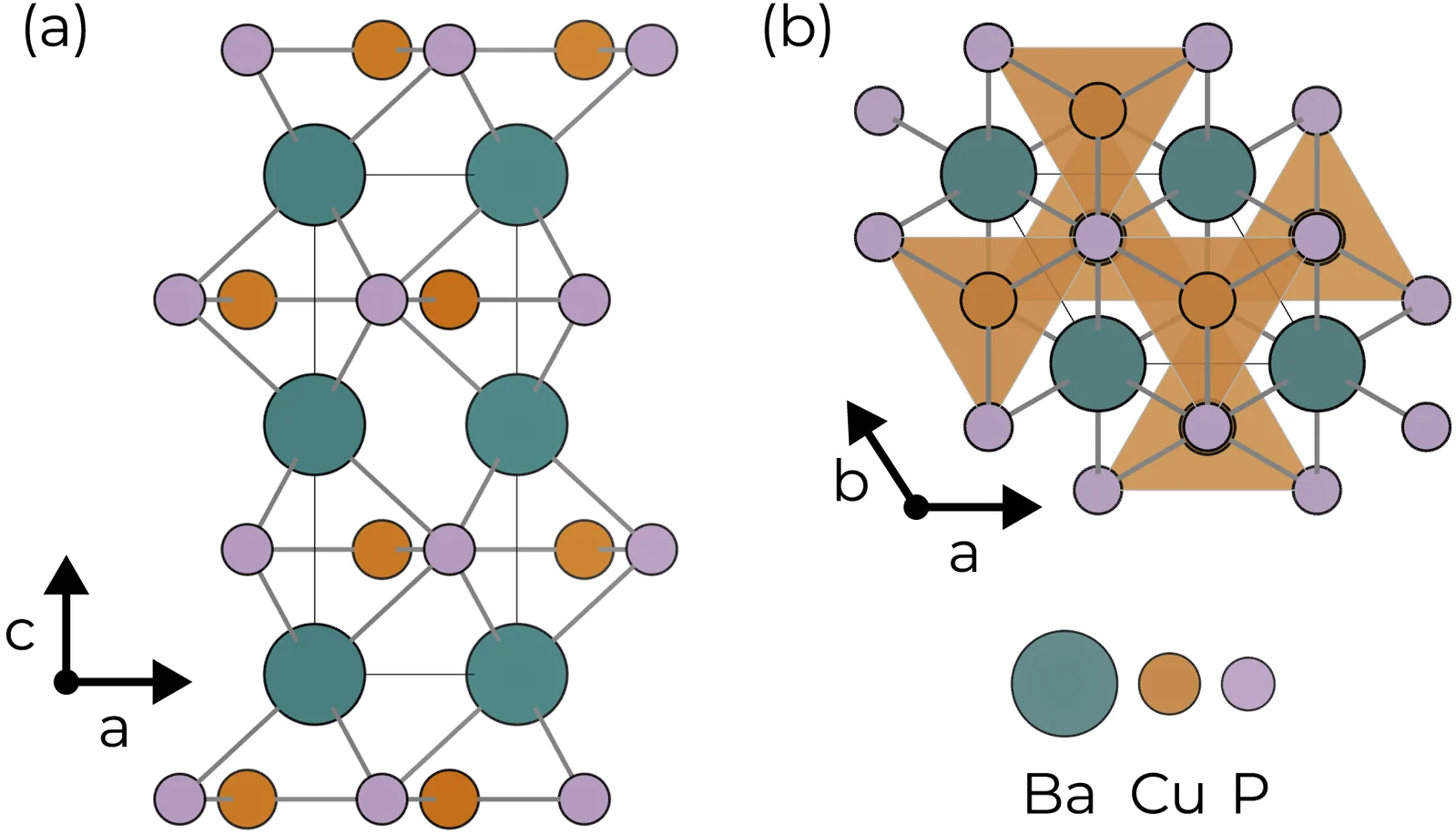
(a) Crystal structure of BaCuP (*P*6_3_/*mmc*) with (b) the corresponding crystal connectivity of the Cu–P layers.

In this work, we investigate the potential of BaCuP as a next-generation metal-phosphide thermoelectric material using density functional theory (DFT) for electronic properties, and machine-learning interatomic potential for advanced lattice thermal conductivity calculations. BaCuP crystallizes in the hexagonal *P*6_3_/*mmc* space group, analogous to other 1-1-1 phosphides such as CaCuP and SrCuP.^24,25^ Its structure consists of hexagonal Cu–P layers separated by Ba cations, as shown in Fig. 1. Notably, SrCuP, CaCuP and CaAgP have been experimentally investigated for thermoelectric applications, reaching a peak *zT* ranging from 0.2 to 0.5.^19,26–29^ Comparatively, BaCuP has received very little attention. Our calculations indicate that adopting Ba (instead of lighter alkaline-earth cations) could help reduce the lattice thermal conductivity while preserving a power factor comparable (even slightly higher) to that of other 1-1-1 phosphides. As a result, BaCuP is predicted to reach a peak *zT* close to 0.69, although its thermoelectric performance is highly anisotropic and strongly limited by the poor out-of-plane transport. We further show that nanostructuring is not a productive route in this material, because it suppresses the carrier transport more strongly than the lattice thermal conductivity. Finally, our results suggest that the carrier concentration required to achieve these high *zT* values may be reached by doping BaCuP with La, which lowers the hole concentration associated with the substantial intrinsic concentration of copper vacancies.

## 2. Computational details

All DFT calculations were performed using the Vienna *ab initio* simulation package (vasp) code.^30,31^ This includes band structure, transport, and defects calculations using the hybrid HSE06 functional,^32^ and DFPT (dielectric constant, harmonic phonon properties) and single-point calculations used for the structures in the machine-learning interatomic potential (MLIP) dataset using GGA PBEsol functional. Plane-wave cutoff and *k*-point grid were converged, resulting in a cutoff of 400 eV and a *k*-point grid of 6 × 6 × 3. HSE06 and PBEsol relaxations of the structure lead to lattice parameters of 4.26 Å and 4.14 Å for the *a*- and *b* – axis and 9.11 Å and 9.18 Å for the *c*-axis, respectively. Both are in good agreement with experimental measurements (*a* = 4.24 Å and *c* = 9.01 Å).^25^

Point defects were modelled using a supercell approach. To minimize interactions between periodic images a supercell of 48 atoms was used to maintain *a* >10 Å distance between periodic images, as generated using the doped package.^33^ The shakenbreak approach^34,35^ was used to identify the ground state defect structures of each defect. The identified ground state structures were further relaxed using a converged 2 × 2 × 2 *k*-point grid. The doped package was then used to post-process the results and to obtain transition level diagrams (TLDs), self-consistent Fermi energies and defect and carrier concentrations. More details about the methodology can be found in the SI. Our defect calculations followed the recently proposed guidelines for reproducibility^36^ and the data are freely available on Zenodo (https://doi.org/10.5281/zenodo.21215101).

Regarding the lattice dynamics properties, we trained an MLIP model from scratch using a dataset of single-point DFT calculations on rattled and distorted structures generated using the calorine code,^37^ and complemented with structures obtained using snapshots of molecular dynamics (MD) simulations performed with the GPUMD software.^38,39^ The dataset comprises a total of 670 structures. The gpumd software was used for the MD runs and for the training of the neuroevolution potential (NEP) model. The convergence of the NEP model was monitored through the evolution of the training and test losses with generation, and its accuracy was further assessed *via* parity plots of the predicted energies, forces, virials and stresses against the corresponding DFT reference values (Fig. S1 and S2). The model was also validated against 2nd and 3rd order finite displacements, for which an excellent agreement was found as seen in the phonon band structure and third-order energies/forces comparison for a subset of structures (Fig. S3a and b). The final model and input files used to generate the model are also provided in the paper-related Zenodo database (https://doi.org/10.5281/zenodo.21215101).

The lattice thermal conductivity was calculated using our NEP model and the homogeneous non-equilibrium molecular dynamics (HNEMD) method.^40,41^ A supercell of 12 288 atoms (a supercell extension of 16 × 16 × 8) and a driving force of *F*_e_ = 2 × 10^−5^ Å^−1^ along the transport direction were used. A Nose–Hoover chain thermostat was applied to control the temperature of the system. For improved signal-to-noise and reproducibility, we used multiple independent HNEMD trajectories for each temperature and transport direction (20 runs), and report the standard error of the mean. We note that at 1400 K only the ten longest trajectories were retained for the final average because of instabilities and memory costs. To assess size effects relevant to nanostructuring, we additionally computed the spectral heat current (SHC),^40,41^ and combined the resulting spectral thermal conductivity from HNEMD with the spectral conductance obtained from short “ballistic” nonequilibrium molecular dynamics (NEMD) simulations employing a pair of local thermostats (source/sink), enabling the reconstruction of the apparent lattice thermal conductivity as a function of length.^41^ By construction, the HNEMD approach includes all possible effects of anharmonicity, allowing lattice thermal conductivity to be calculated beyond the traditional third-order approximation.

Finally, charge transport calculations were performed using the Boltzmann transport equation, as implemented in the AMSET software.^42^ The contributions due to polar-optical phonons (POP), acoustic deformation potential (ADP) and impurity (IMP) scattering were included. No piezoelectric response was observed in BaCuP, leading to no piezoelectric (PIE) scattering. Bipolar transport is included in all the transport properties reported here: AMSET constructs the spectral conductivity as a sum over all bands lying within the Fermi–Dirac window and evaluates the Onsager coefficients with a single Fermi level, so that contributions from thermally excited minority carriers arise automatically once both carrier types are populated. In our calculations this window spans 1.72–4.99 eV while the conduction-band minimum lies at 4.04 eV, so the conduction band is included at all temperatures considered. To assess the effect of nanostructuring on the carrier transport, an additional boundary scattering rate *v*_nk_/*L* was included for every electronic state, where *v*_nk_ is the group velocity of the state and *L* the mean free path. The transport coefficients were re-evaluated with these modified lifetimes at each length. The results are shown in the Nanostructuring section. The charge transport calculations were performed without spin–orbit coupling, as only a negligible effect was observed on the mobility (see Table S1). We note that a small underestimation of the charge transport properties is expected, as AMSET is based on relaxation time approximations.^43–45^ Alternative implementations based on the iterative (“exact”) solution of the Boltzmann transport equation typically require a finite GGA band gap, which is not the case for BaCuP, preventing this route here. The corresponding settings, inputs and *k*-grid convergence testing (Fig. S4) for the AMSET calculations are provided in the SI. For the IMP scattering, AMSET evaluates the concentration of ionised scattering centres as *n*_ii_ = *C* (*n*_0_ − *p*_0_)/*q*, where (*n*_0_ − *p*_0_) is the concentration of excess donors/acceptors, *q* the defect charge and *C* a compensation factor. The latter accounts for the fact that donors and acceptors of opposite sign both scatter carriers, while partially cancelling one another in the net carrier concentration. We retain the default value *C* = 2, corresponding to a donor-to-acceptor ratio *N*_D_/*N*_A_ ≈ 1/3, for all doping scenarios. A fully self-consistent treatment would require *C* to be evaluated from the defect concentrations at every carrier concentration and temperature and fed back into the transport calculation, whereas a single value is applied across the whole grid in AMSET. Keeping *C* fixed also ensures that the three doping scenarios are compared on an identical footing. The implications of this approximation are quantified in the electronic transport section. Throughout this work, averaged transport properties are obtained by taking the arithmetic mean of the 3 directions of *σ*, *S*, *κ*_e_ and *κ*_l_, and evaluating *zT* from these averaged quantities. For the hexagonal structure of BaCuP this corresponds to (2*Q*_a_ + *Q*_c_)/3, since *Q*_a_ = *Q*_b_.

## 3. Electronic structure

**Fig. 2:**
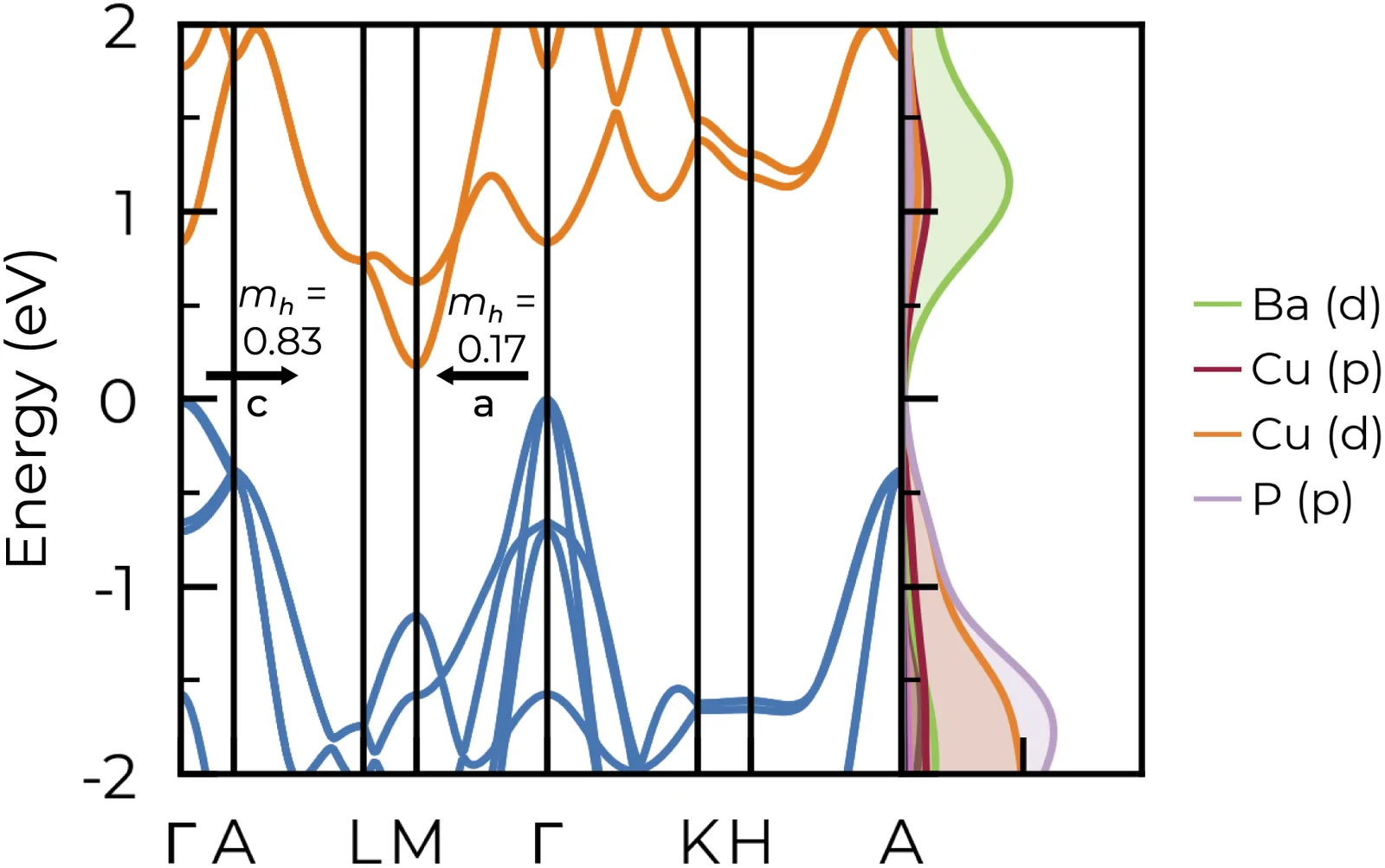
Electronic band structure of BaCuP and the corresponding density of states (DOS). Plotted using sumo.^47^

Fig. 2 shows the hybrid DFT band structure of BaCuP together with the associated density of states (DOS). BaCuP exhibits a small indirect band gap of around 0.19 eV, and a larger direct gap of 0.846 eV. The highly dispersive valence band maximum (VBM) arises solely from interactions between P p and Cu d orbitals, as also observed in other 1-1-1 phosphides,^46^ and leads to low conductivity effective masses of around 0.17 m_e_ (in-plane) and 0.83 m_e_ (out-of-plane). These values are similar to those previously reported,^46^ revealing a strong anisotropy between intralayer and interlayer transport. We also observe a very limited effect of SOC, with only a small split-off of around 20 meV (see Fig. S5), no significant changes in the band curvature and conductivity effective masses (0.20 m_e_ and 0.83 m_e_ with SOC) or the hole mobility (Table S1).

## 4. Defect chemistry

### 4.1. Intrinsic defects

**Fig. 3:**
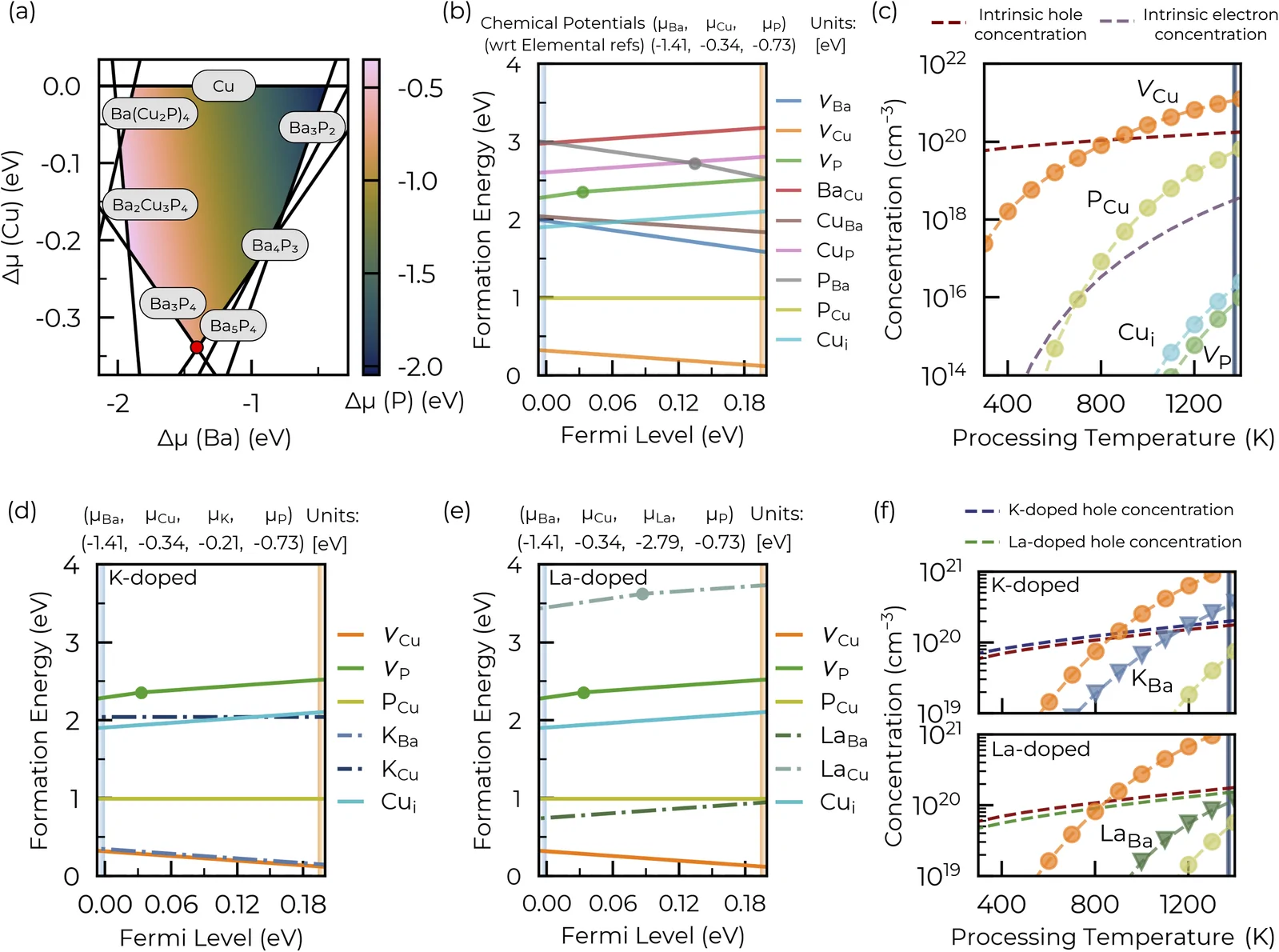
(a) Accessible chemical potential range for the formation of BaCuP. The red dot represents the best p-type conditions (Cu-poor). (b) Transition level diagram of intrinsic point defects in BaCuP under Cu-poor (p-type) conditions. (c) Intrinsic defect and hole concentrations as a function of the processing temperature. The blue line represents the experimental annealing temperature, as mentioned in ref. 25. (d) Transition level diagram of K-doped and (e) La-doped BaCuP in Cu-poor (p-type) conditions. (f) Corresponding defect and hole concentrations as a function of the processing temperature for K- and La-doped BaCuP. The dashed red line represents the intrinsic hole concentration.

Fig. 3(a) illustrates the HSE06-calculated accessible range of chemical potentials for BaCuP. The shaded region represents the stability range of chemical potentials (Δ*µ*_Ba_, Δ*µ*_Cu_, and Δ*µ*_P_) imposed by the formation of BaCuP and constrained by multiple competing phases, namely Cu, Ba_3_P_2_, Ba_4_P_3_, Ba_5_P_4_, Ba_3_P_4_, Ba_2_Cu_3_P_4_, and Ba(Cu_2_P)_4_.^48^ Within these boundaries, the red dot in Fig. 3(a) marks the selected p-type growth conditions (“Cu-poor”) used in this study.

The transition level diagram of intrinsic BaCuP is shown in Fig. 3(b). We note that Ba_i_, P_i_, and Ba_P_ were found to have very high formation energies and do not appear in the plot. The lowest-energy defect across the full Fermi level range is the copper vacancy (*V*_Cu_), as is commonly observed in these phosphides.^17,20,27,28^ This defect is the main contributor to the high carrier concentration observed in BaCuP, as shown in Fig. 3(c). In this plot, the intrinsic hole concentration (in red) results from annealing at 1370 K followed by quenching to the different processing temperatures. This annealing temperature was chosen following the experimental procedure mentioned in ref. 25. The P_Cu_ antisite is the next lowest energy defect and remains neutral across the full Fermi level range, as this charge state is stabilised by a P–P dimer (Fig. S6). Finally, Cu_i_ and *V*_P_ are the first compensating defects, but both remain relatively high in energy. As a result, the concentrations of these two defect species are low and compensation is limited. As shown in Fig. S7, this results in an intrinsic Fermi level already pushed into the valence band, indicating degenerate semiconductor behaviour. This is also hinted at by the high intrinsic hole carrier concentration achievable in BaCuP. The electron (minority carrier) concentration, also shown in Fig. 3(c), remains orders of magnitude below the hole concentration across the full temperature range considered, confirming that BaCuP remains strongly p-type even at elevated temperatures. The absence of low energy compensating defects also provides a large doping window (around 1.9 eV), which could favour extrinsic doping. As thermoelectric materials usually require specific carrier concentrations to achieve their full potential, we note that the hole concentration in BaCuP could be readily tuned by changing the growth conditions, since both the main donor and acceptor defects are Cu-related. For example, this could be achieved by moving from the most p-type conditions (shown by the red dot in Fig. 3(b)) to less Cu-poor conditions. In this study, we consider another possible route *via* the use of extrinsic dopants.

### 4.2. K- & La-doped BaCuP

In this work, we considered two different dopants on the Ba site, namely K and La. As shown by the DOS (Fig. 2), the Ba site is the most suitable substitution site for preserving the favourable electronic properties of the VBM, which is dominated by Cu–P interactions. On the one hand, doping with K^+^ on the Ba^2+^ site is expected to introduce additional holes into the system, which is typically beneficial for improving the conductivity. On the other hand, La^3+^ on the Ba^2+^ site could slightly reduce the carrier concentration, which may help to obtain a higher Seebeck coefficient. In both cases, the introduction of another relatively heavy extrinsic element could also help reduce the lattice thermal conductivity.^49,50^ Nevertheless, the exact effect of these dopants on the lattice thermal conductivity is left for future work.

Fig. 3(d) and (e) show the TLD of K-doped and La-doped BaCuP, respectively. For both dopants, we consider Cu-poor growth conditions. The K substitution on the Ba site has a low formation energy, only slightly higher than that of the copper vacancy. For La, the substitution is higher in energy, but still favourable. This can be explained by the accessible chemical potential range in both cases, which allows a relatively high *µ*_K_ under Cu-poor conditions, while this is not the case for La. For both dopants, substitution on the Ba site is far more favourable than substitution on either the Cu or P sites. The K_P_ and La_P_ are not shown in the TLDs because of their much higher formation energies.

In terms of carrier concentration (see Fig. 3(f)), the copper vacancy remains the dominant defect in both cases, but K_Ba_ and La_Ba_ closely follow, reaching significant defect concentrations. As expected, this leads to a slight increase in hole concentration for K doping and a slight decrease for La doping. This extends the available range for tuning the carrier concentration and, therefore provides additional flexibility for achieving the optimum *zT* in BaCuP. For both doping scenarios, *n*_e_ remains at least three orders of magnitude below *n*_h_ up to 900 K, and only approaches the percent level near the annealing temperature (∼1370 K), as seen in Fig. S8.

## 5. Thermoelectric properties

### 5.1. Lattice thermal conductivity

**Fig. 4:**
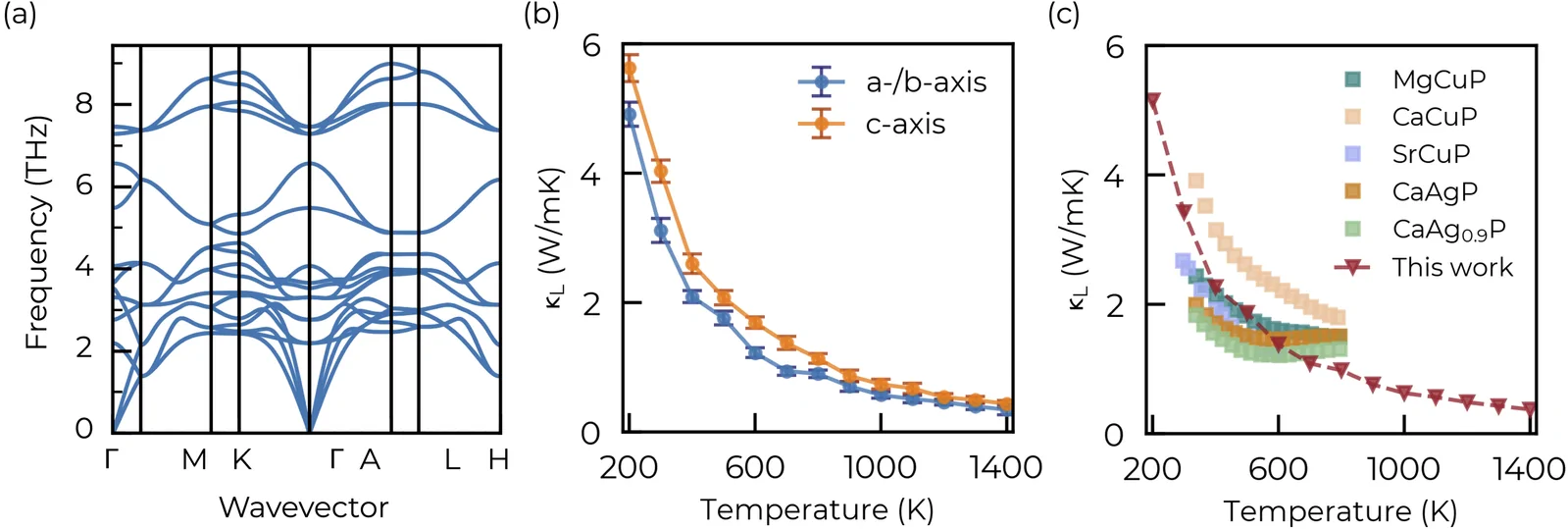
(a) Phonon band structure (plotted using sumo^47^) (b) Lattice thermal conductivity against temperature for the in-plane (*a*-/*b*-axis) and out-of-plane directions. The standard error of the mean (SEM) is represented as an error bar. (c) Average lattice thermal conductivity compared with experimental measurements of other 1-1-1 phosphides.^19,26–28^

Fig. 4(a) shows the phonon band structure of BaCuP, as computed with the MLIP approach, and confirms its dynamical stability. Interestingly, despite its relatively simple crystal structure, BaCuP exhibits relatively low group velocity but also large Grüneisen parameters, suggesting substantial anharmonicity (see Fig. S9). This highlights the need to go beyond conventional lattice thermal conductivity calculations based only on third-order phonon interactions within the Boltzmann transport formalism. To account for these higher-order anharmonic effects, we therefore go beyond the conventional BTE for thermal transport and employ the linear response HNEMD method.^40^

The lattice thermal conductivity computed using HNEMD is shown in Fig. 4(b), where a small anisotropy is again observed between the in-plane and out-of-plane directions, although much less pronounced than for the effective masses. The error bars represent the standard error of the mean (SEM), with each point corresponding to the cumulative average of 20 runs of 20 ns. The number of runs and the simulation length were chosen following multiple convergence tests to ensure sufficiently steady behaviour of the curves, especially given the relatively low lattice thermal conductivity values here. We note the exception of the 1400 K data points, for which the MD simulations were more challenging and for which only the 10 longest runs were retained. Compared with phono3py calculations^51,52^ based on the third-order BTE (see Fig. S10), our HNEMD results give a lower lattice thermal conductivity, which could be attributed to the importance of higher-order anharmonic terms, as also suggested by the large Grüneisen parameters. The low lattice thermal conductivity of BaCuP can therefore be attributed to relatively small group velocities, but more importantly to strong phonon scattering.

The average lattice thermal conductivity of BaCuP (arithmetic mean of the directional values) is further compared with experimental values reported for other 1-1-1 phosphides. The temperature dependence is very similar to that observed in CaCuP and SrCuP, which share the same crystal structure. The lower lattice thermal conductivity predicted here for BaCuP compared to CaCuP is likely related to the heavier Ba cation relative to Ca. A similar difference exists when comparing our results with theoretical predictions on CaCuP.^53^ However, SrCuP appears to exhibit a lower lattice thermal conductivity than our predicted values, which may seem surprising. Direct comparison with experimental measurements is not straightforward, as our predictions only include phonon–phonon interactions in a “perfect” crystal, whereas 1-1-1 phosphides are typically synthesised as pressed pellets,^19,20,26–28^ for which surfaces and interfaces can introduce additional scattering sources. In addition, pressed pellets typically reach only a fraction of the theoretical density, which can further reduce the measured *κ*_l_ relative to our single-crystal prediction. This is also relevant for the electronic transport properties, as discussed in the next section. MgCuP adopts a different and more complex crystal structure, which is likely responsible for its lower lattice thermal conductivity at low temperature, although this behaviour appears to flatten rapidly with increasing temperature. A similar trend is observed for CaAgP, for which the lattice thermal conductivity is the lowest at low temperature but quickly reaches a plateau around 500 K. This has been attributed to an increasing bipolar contribution to the total thermal conductivity.^19,27^ Current experimental methods tend to extract a mix of lattice and bipolar thermal conductivity instead of the pure lattice thermal conductivity.

### 5.2. Electronic transport properties

**Fig. 5:**
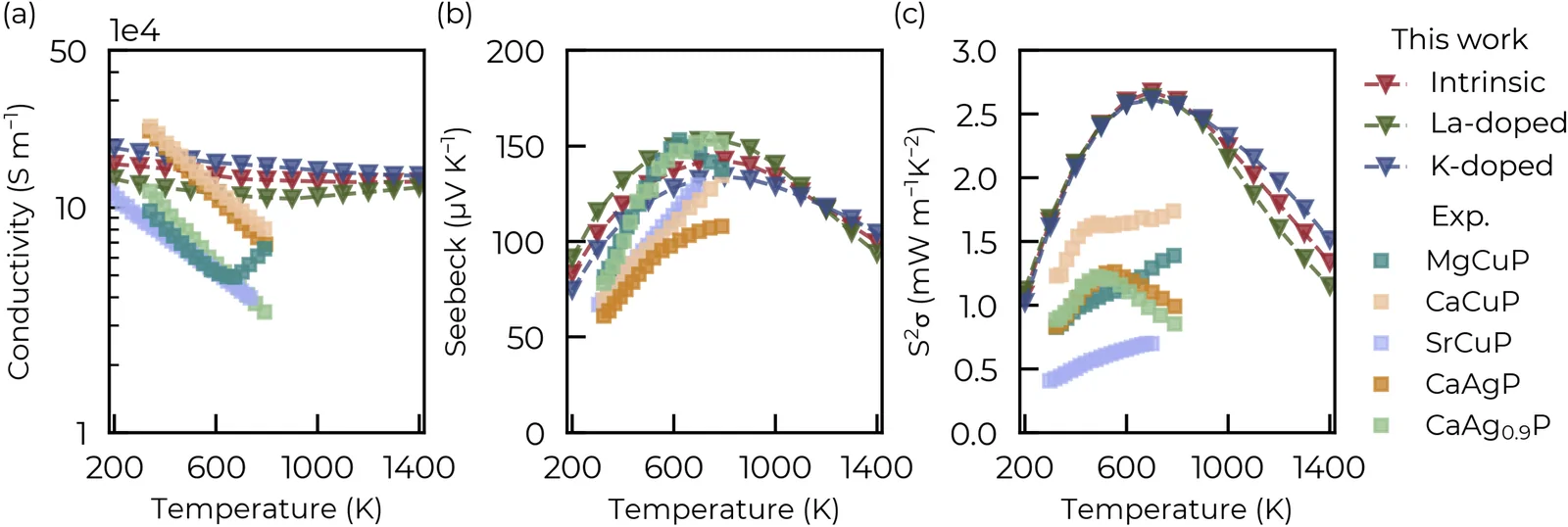
(a) Hole conductivity, (b) Seebeck coefficient and (c) power factor of intrinsic (red triangle), La-doped (green triangle) and K-doped (blue triangle) BaCuP against T. Experimental measurements on other 1-1-1 phosphides are shown for comparison^19,26–28^

Starting with the hole conductivity, we predict a conductivity around 15 × 10^4^ S m^−1^ in the range of considered temperatures, as shown in Fig. 5(a). Only a slight decrease of the conductivity with increasing temperature is observed. As temperature increases, the carrier concentration tends to rise (see Fig. 3(c) and (f)), while the mobility decreases due to enhanced scattering, arising both from the larger carrier concentration and increased phonon scattering. As expected, the highest conductivity is obtained for K-doped BaCuP, which exhibits the largest hole concentration, whereas La-doped BaCuP shows a lower conductivity. Compared with other 1-1-1 phosphides, the decrease observed in the conductivity of BaCuP is less pronounced. This difference may arise from several factors, including additional scattering mechanisms such as grain boundary or surface scattering, which are more prevalent in experimentally synthesised pressed pellets than in the ideal crystalline compounds considered in our predictions. This difference of slope has already been observed in a theoretical study on CaAgP.^54^ The sudden change in the conductivity of MgCuP is linked to bipolar transport at temperatures where electron conductivity begins to make a significant contribution to the total conductivity.^27^

The Seebeck coefficient (Fig. 5(b)) is very similar to those experimentally reported for other 1-1-1 phosphides, with values peaking at around 150 µV K^−1^ in the 600–900 K range. Here, the highest Seebeck coefficient is obtained for La-doped BaCuP, due to the lower carrier concentration. Finally, the power factor is predicted to be slightly higher in BaCuP than in other 1-1-1 phosphides, as shown in Fig. 5(c). Once again, the discrepancy between experimental measurements performed on pellets and the predicted results obtained for single crystals may help explain this difference. The calculated power factor of BaCuP remains larger than that of CaCuP predicted using a similar theoretical methodology.^53^ In any case, the power factor of all 1-1-1 phosphides is well below that of standard thermoelectric materials such as half-Heusler materials.^55,56^ This remains one of the main bottlenecks of metal phosphides for practical TE applications.^17^ Finally, the electrical contribution to the thermal conductivity (*κ*_e_) is shown in Fig. S11. As the doping scenario varies, the trend of the electronic thermal conductivity is similar to that of the conductivity, both being directly linked by the Lorenz number.

Unlike K_Ba_^−^, which is an acceptor of the same sign as the dominant V_Cu_^−^, the La_Ba_^+^ donor introduces charged centres of opposite sign, so that La doping reduces the hole concentration towards an ideal value. But the compensated holes come at the cost of a larger total density of ionised scattering centres. Based on our defect calculations, La doping corresponds to a compensation ratio *N*_D_/*N*_A_ of the order of 55–65% (close to *C* ≈ 4) across the operating temperature range, whereas the intrinsic and K-doped cases remain essentially uncompensated with all significant charged defects carrying the same sign. Fig. S12 shows the effect of varying the compensation factor over the full plausible range. Increasing *C* raises the IMP scattering rate and lowers both the mobility and the electrical conductivity, but it simultaneously increases the Seebeck coefficient and reduces the electronic thermal conductivity by a comparable amount. The conductivity is reduced simply because a larger density of charged centres shortens the relaxation time of every carrier. The Seebeck coefficient, by contrast, is set by the energy derivative of the spectral conductivity at the Fermi level rather than by its magnitude, and since ionised impurities scatter the slow carriers close to the Fermi level much more strongly than the fast ones, they steepen this energy dependence and slightly raise *S* while *σ* and *κ*_e_ both fall. These contributions largely cancel in *zT*, which varies by less than 5% over the whole range of *C* considered. Adopting the compensation factor appropriate to each scenario therefore leaves our conclusions unchanged, with La-doped BaCuP retaining the highest *zT* at all temperatures considered.

A known limitation of metal phosphides as thermoelectrics is the presence of important bipolar contributions,^19,27^ arising from a combination of small band gaps and hole concentrations that are insufficiently high to suppress minority carrier excitation. Bipolar transport is included in all of the transport properties reported here. To quantify this contribution we repeated the calculations with the band gap opened to 2 eV using a scissor operator, keeping all other settings identical, which suppresses the minority carriers (Fig. S13). The effect is far from negligible: relative to the scissored calculation, minority carriers reduce *S* by 5% and raise *κ*_e_ by 17% at 800 K (rising to 18% and 36% at 1000 K). The electronic thermal conductivity is far more sensitive to these minority carriers than the conductivity, because *κ*_e_ weights each carrier by the square of its energy separation from the Fermi level, whereas *σ* counts every carrier equally. Bipolar conduction is therefore a dominant factor in limiting the high-temperature performance of BaCuP, and partly explains both the maximum in *zT*(*T*) near 800 K and its decline above.

### 5.3. Figure of merit *zT*

**Fig. 6:**
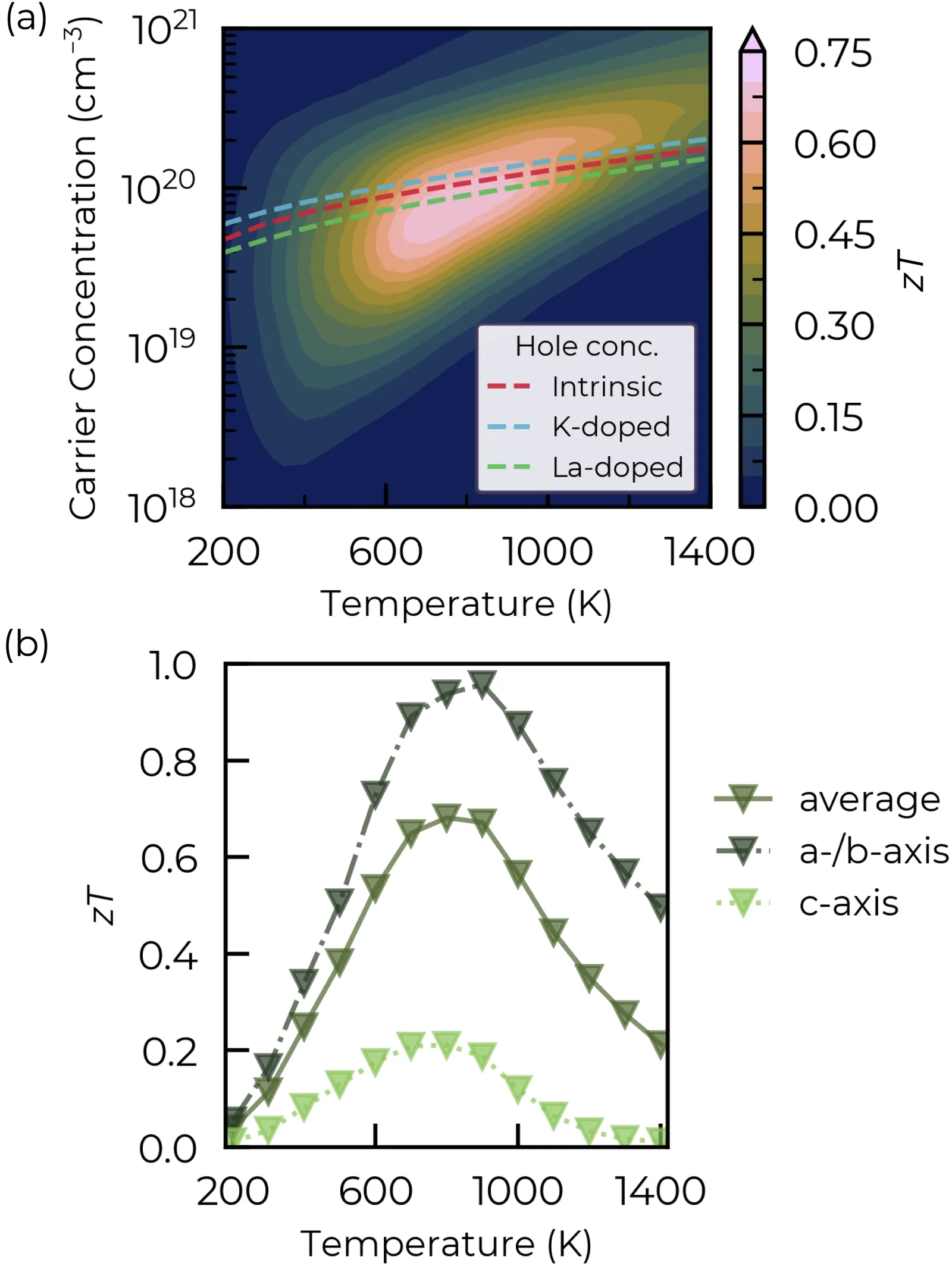
(a) Map of the achievable average *zT* as a function of temperature and carrier concentration in BaCuP. The dashed lines corresponds to the hole concentration attainable at the corresponding temperature for intrinsic, K-doped and La-doped BaCuP. (b) Average, in-plane (*a*-/*b*-axis) and out-of-plane achievable *zT versus* temperature in La-doped BaCuP.

Fig. 6(a) shows a map of the achievable *zT* as a function of temperature and carrier concentration. A directionally averaged (arithmetic mean) peak *zT* of 0.69 is achievable at 800 K for a carrier concentration of 8 × 10^19^ cm^−3^. This map reveals that the carrier concentrations obtained under intrinsic conditions, and therefore also in K-doped BaCuP, are slightly too high to achieve the best performance in BaCuP. In contrast, the hole concentration obtained for La-doped BaCuP under Cu-poor conditions at 800 K lies close to this optimum, so that La-doped BaCuP reaches within 1% of the maximum achievable *zT*. We note that changing the growth conditions to less Cu-poor conditions could also allow intrinsic BaCuP, as well as K-doped BaCuP, to reach the peak *zT*.

Fig. 6(b) demonstrates the strong anisotropy of *zT* in La-doped BaCuP. While the in-plane direction, within the Cu–P layers, could achieve a *zT* close to 1, the out-of-plane *zT* is much lower and only slightly exceeds 0.2. This pronounced anisotropy in *zT* arises primarily from the anisotropy in the electronic transport properties, as exemplified by the large difference in conductivity effective mass, and is further reinforced by the (much smaller) anisotropy in the lattice thermal conductivity. Improving the thermoelectric performance of BaCuP could therefore require enhancing this weaker *c*-axis transport direction. The directionally averaged *zT* of the three doping scenarios is compared in Fig. S14, and the corresponding in-plane and out-of-plane values for the intrinsic and K-doped scenarios are given in Fig. S15. We also note that the directional average considered in this work should be compared with caution to the effective property of a polycrystal and comparison with experiment should be regarded as indicative rather than quantitative. In the case of these phosphides, most measurements are made on pressed pellets, which are porous, may be textured rather than randomly oriented, and contain grain boundaries that scatter both carriers and phonons, none of which is captured by our defect-free single-crystal calculations.

**Fig. 7:**
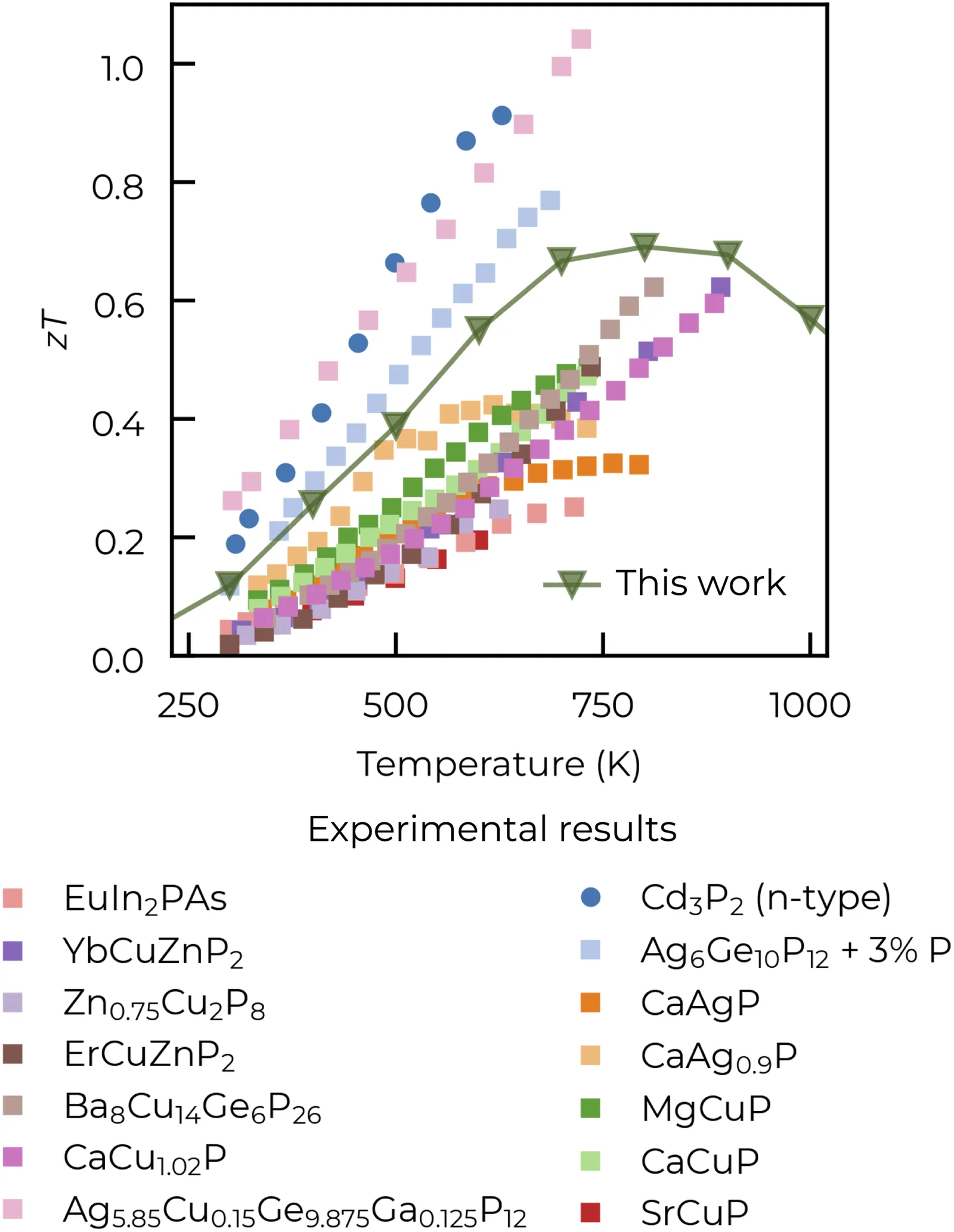
Computed average figure of merit *zT* of La-doped BaCuP against temperature (green triangle). Experimental *zT* for different phosphides (EuIn_2_PAs,^57^ YbCuZnP_2_,^58^ Zn_0.75_Cu_2_P_8_,^59^ ErCuZnP_2_,^60^ Ba_8_Cu_14_Ge_6_P_26_,^61^ CaCuP and CaCu_1.02_P,^27,28^ Ag_5.85_Cu_0.15_Ge_9.875_Ga_0.125_P_12_,^23^ Cd_3_P_2_,^21^ Ag_6_Ge_10_P_12_ + 3% P,^18^ CaAgP and CaAg_0.9_P,^19^ MgCuP,^27^ SrCuP^26^) are shown for comparison.

With a peak average *zT* of 0.69 at 800 K, La-doped BaCuP already presents the highest figure of merit among the 1-1-1 phosphides but also surpasses most of the TE phosphides, as shown in Fig. 7. Only (co)-doped Ag_6_Ge_10_P_12_ possess a higher *zT* value due to its complex band structure and high power factor, and Cd_3_P_2_ (n-type) which possesses an extremely high mobility.

### 5.4. Nanostructuring

**Fig. 8:**
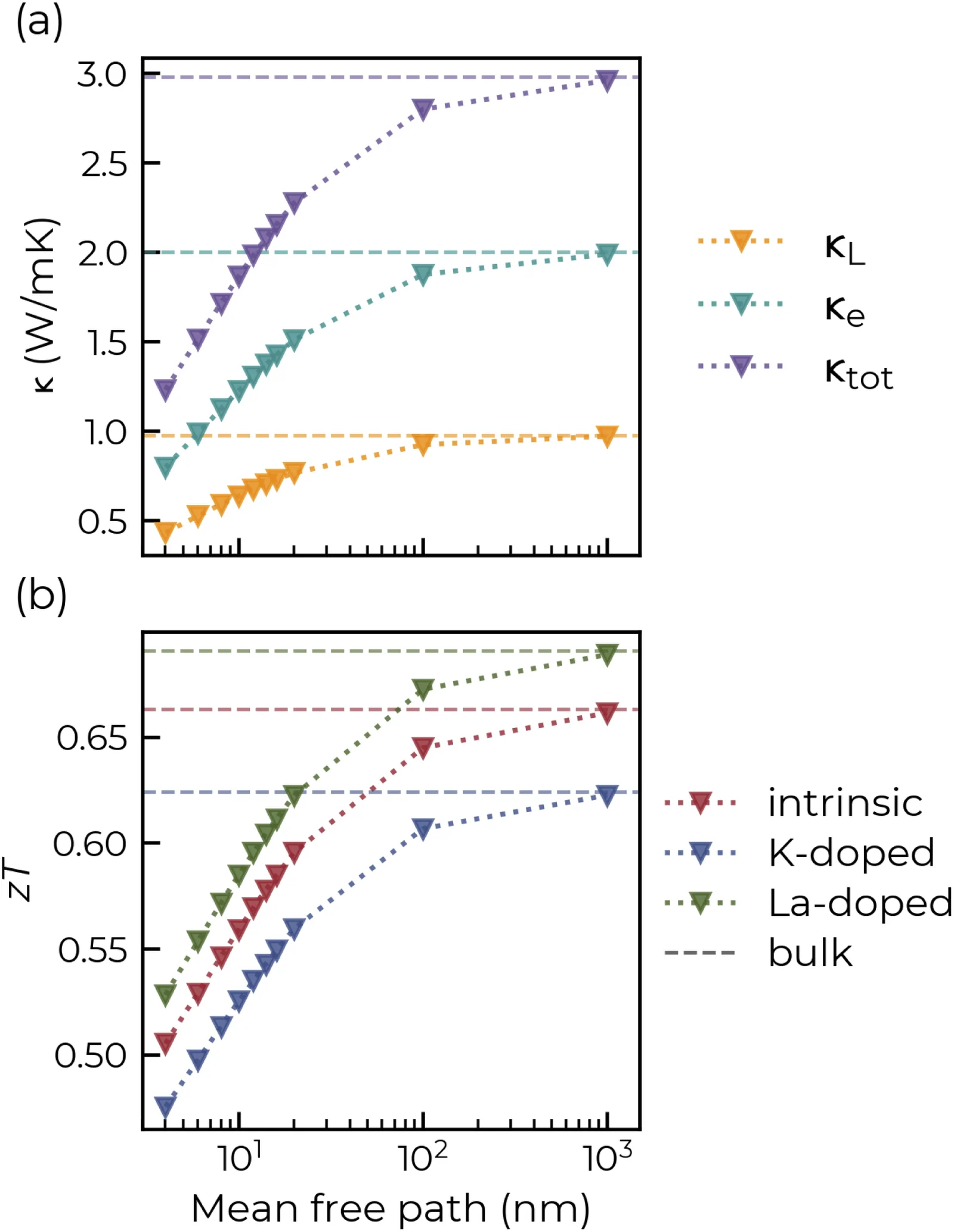
(a) Directionally averaged lattice (*κ*_l_), electronic (*κ*_e_) and total (*κ*_tot_) thermal conductivity of La-doped BaCuP against the mean free path at 800 K. (b) Directionally averaged figure of merit *zT* against the same mean free path at 800 K for the intrinsic, K-doped and La-doped scenarios. The dashed horizontal lines indicate the corresponding bulk values.

One common method to improve the *zT* of TE materials is nanostructuring, which increases the scattering of phonons and reduces the lattice thermal conductivity. Fig. 8(a) shows the decrease of *κ*_l_ with decreasing mean free path at 800 K (the results for other temperatures can be seen in Fig. S16). The same boundary scattering also limits the carriers, so that the electronic contribution is reduced as well, and in this case more strongly: between the bulk and a mean free path of 4 nm, *κ*_l_ decreases by 0.55 W m^−1^ K^−1^ whereas *κ*_e_ decreases by 1.20 W m^−1^ K^−1^, so that under such heavy nanostructuring the total thermal conductivity is divided by a factor of ≈2.4.

However, a balance is important to find, as this reduction of the mean free path also degrades the quantities entering the numerator of *zT*. We account for this explicitly by recomputing *σ*, *S* and *κ*_e_ at each length scale (Fig. S17). The additional carrier scattering reduces the hole mobility, from 75 to 28 cm^2^ V^−1^ s^−1^ between the bulk and *L* = 4 nm, and lowers the Seebeck coefficient as well. Over the same range the power factor falls by a factor of ≈3.2, faster than the total thermal conductivity, and *zT* therefore decreases monotonically. As shown in Fig. 8(b), nanostructuring lowers the figure of merit at every length scale considered, from 0.69 in the bulk to 0.62 at 20 nm and 0.53 at 4 nm in the case of La-doped BaCuP. Nanostructuring is only beneficial when it suppresses phonon transport more than carrier transport. This is not the case here: at every length scale considered, reducing the characteristic dimension suppresses the electrical conductivity more strongly than the lattice thermal conductivity, so that the carriers are in fact more sensitive to the reduced dimension than the phonons. BaCuP is in this sense already close to a “phonon glass” in its bulk form, and the low lattice thermal conductivity that makes it attractive as a thermoelectric is precisely what leaves little room for further gains from nanostructuring. Therefore, a similar situation can be expected across the 1-1-1 phosphides, which combine intrinsically low lattice thermal conductivities with relatively mobile carriers.

## 6. Conclusion

In summary, we have identified BaCuP as a promising thermoelectric material within the family of 1-1-1 phosphides, using first-principles calculations and machine-learning. We show that BaCuP combines a favourable Cu–P derived valence band for hole transport with a comparatively low lattice thermal conductivity, the latter being reduced by both the heavy Ba cation and strong anharmonic phonon scattering. Our HNEMD calculations further indicate that higher-order anharmonic effects play an important role in this material and should be considered when assessing thermal transport in related phosphides. From the defect chemistry perspective, BaCuP is predicted to be an intrinsically degenerate p-type conductor due to the dominance of copper vacancies under Cu-poor conditions. This intrinsic behaviour is advantageous for conductivity, but the resulting hole concentration is slightly above the optimum for maximising *zT*. Among the dopants considered here, La on the Ba site appears to provide the most effective route to tune the carrier concentration toward more favourable values, while preserving the electronic structure responsible for good transport within the Cu–P layers. Although the compensating La_Ba_^+^ donors do increase the density of ionised scattering centres, we find this to have only a marginal effect on the predicted performance. As a result, La-doped BaCuP is predicted to reach a peak average *zT* of about 0.69 at 800 K, making it one of the most promising 1-1-1 phosphide thermoelectrics reported to date. The strong anisotropy of transport remains the main limitation, with excellent in-plane performance but much poorer out-of-plane response. This also points to clear strategies for future improvement, including directional control in synthesis, and defect and growth-condition engineering. Nanostructuring, by contrast, is not a viable route here, since reducing the mean free path suppresses the carrier transport more strongly than the lattice thermal conductivity. Overall, our results establish BaCuP as an intriguing platform for the future design of efficient phosphide-based thermoelectrics.

## Author contributions

R. C.: conceptualisation, methodology, investigation, formal analysis, visualisation, writing – original draft. P. P. R.: investigation – review & editing. A. G. S.: methodology, supervision – review & editing. D. O. S.: conceptualisation, supervision, funding acquisition – review & editing.

## Conflicts of interest

There are no conflicts to declare.

## Supplementary Material

TA-014-D6TA05718G-s001

## Data Availability

The data supporting this article, including the relaxed structure, VASP and AMSET inputs and outputs, defect thermodynamics dictionaries, and the NEP model with the associated GPUMD inputs, are available on Zenodo at https://doi.org/10.5281/zenodo.21215101. Supplementary information (SI): the validation of the machine-learning interatomic potential, convergence and spin-orbit coupling tests for the transport calculations, additional defect and carrier-concentration results, the Grüneisen parameters and a phono3py-HNEMD comparison, and analyses of the ionised-impurity compensation factor, the bipolar contribution, the directional figures of merit, and the effect of nanostructuring. See DOI: https://doi.org/10.1039/d6ta05718g.
