# Supplementary material for: Optimizing the thermoelectric performance of the earth-abundant layered phosphide BaCuP through defect engineering
Source: J Mater Chem A Mater. 2026 Aug 28;14(59):40794–804. doi: 10.1039/d6ta05718g (PMC13548382; doi:10.1039/d6ta05718g)
Supplement: Supplementary file 1 [file TA-014-D6TA05718G-s001.pdf]

Supplementary Information (SI) for Journal of Materials Chemistry A.
This journal is © The Royal Society of Chemistry 2026

Optimizing the Thermoelectric Performance of the Earth-Abundant Layered Phosphide BaCuP through Defect Engineering – Supporting Information

Romain Claes [1,*] Peter P. Russell [1] Alexander G. Squires [1] and David O. Scanlon [1,†]
[1] *School of Chemistry, University of Birmingham, Edgbaston, Birmingham, B15 2TT, UK*

[*] r.claes@bham.ac.uk
[†] d.o.scanlon@bham.ac.uk

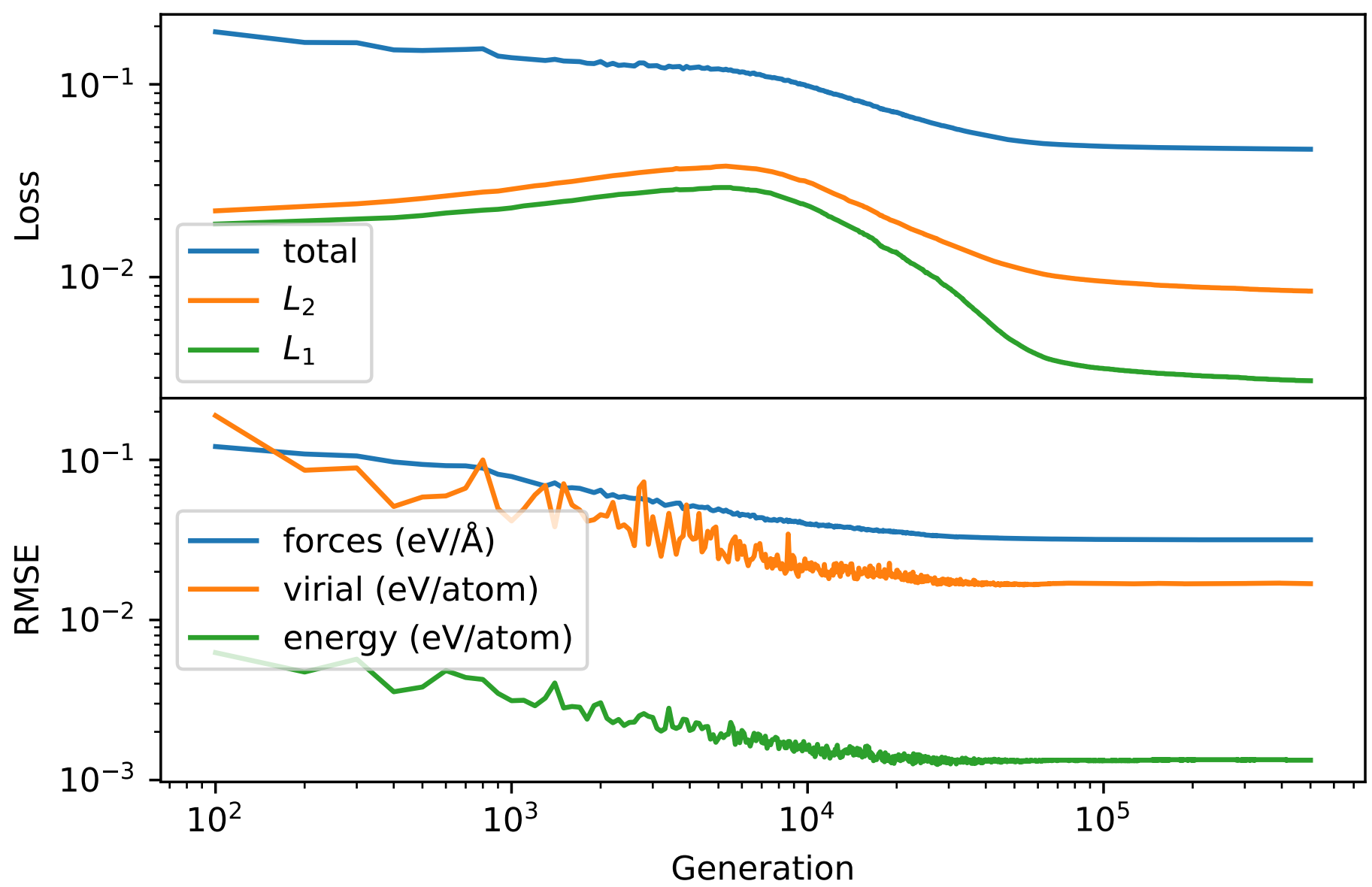

FIG. 1. Total loss function, the L1 and L2 regularization part of the loss function and the corresponding RMSE (root mean squared error) for the full training set of the NEP model.

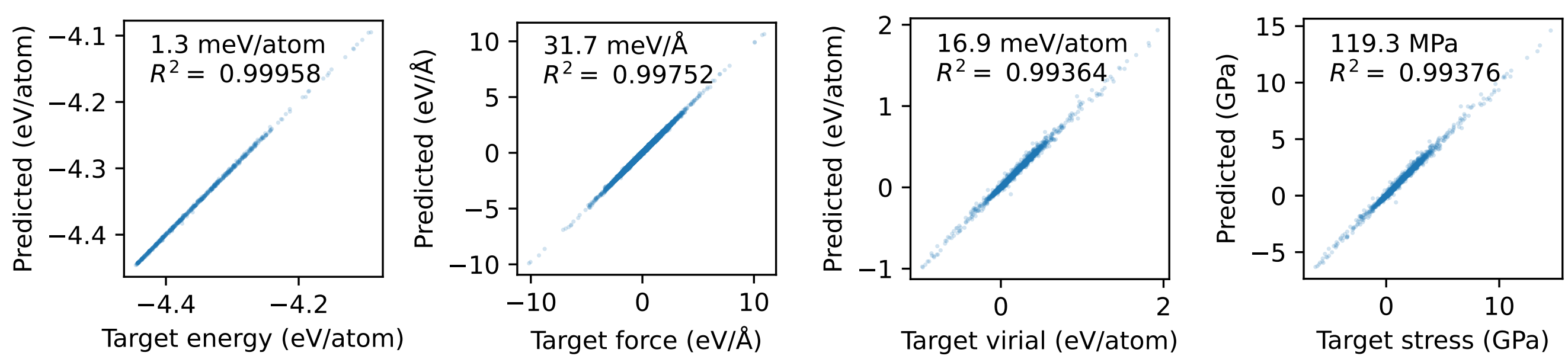

FIG. 2. Parity data of the predicted (with the NEP model) and target (computed with DFT) energies, forces, virials and stresses.

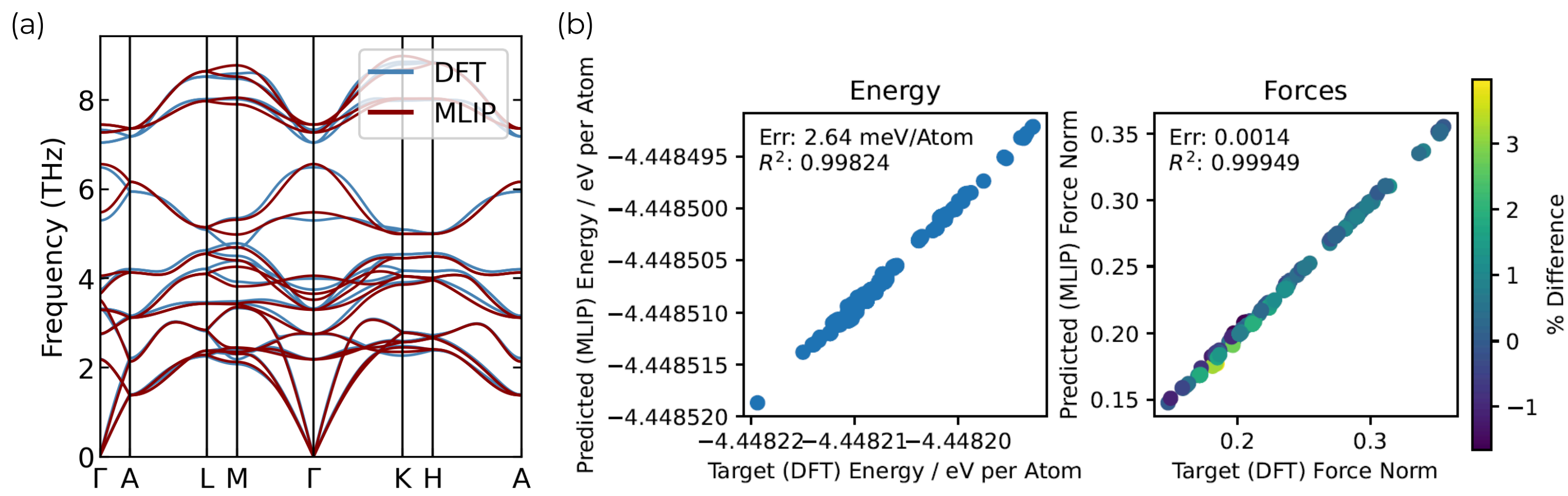

FIG. 3. (a) Comparison of the phonon band structure (harmonic approximation ; 2nd order) obtained with the NEP model (MLIP) and DFT. (b) 3rd order energies/forces comparison for a subset of structures generated with phono3py.

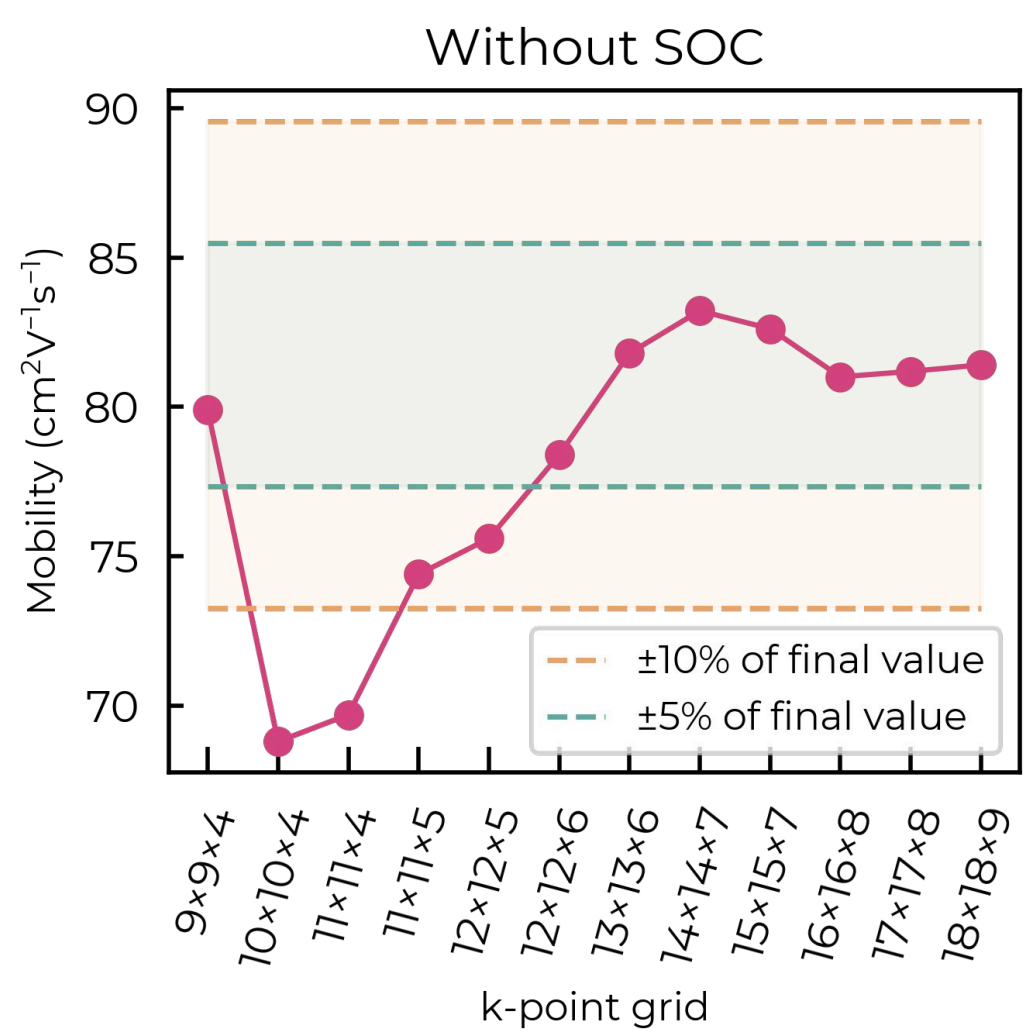

FIG. 4. Mobility convergence with respect to the **k**-point grid using a constant relaxation time of 10^{-14} s under the constant relaxation time approximation (CRTA). The interpolation factors were converged independently for each calculation. Convergence is assumed with a $12 \times 12 \times 6$ **k**-point grid.

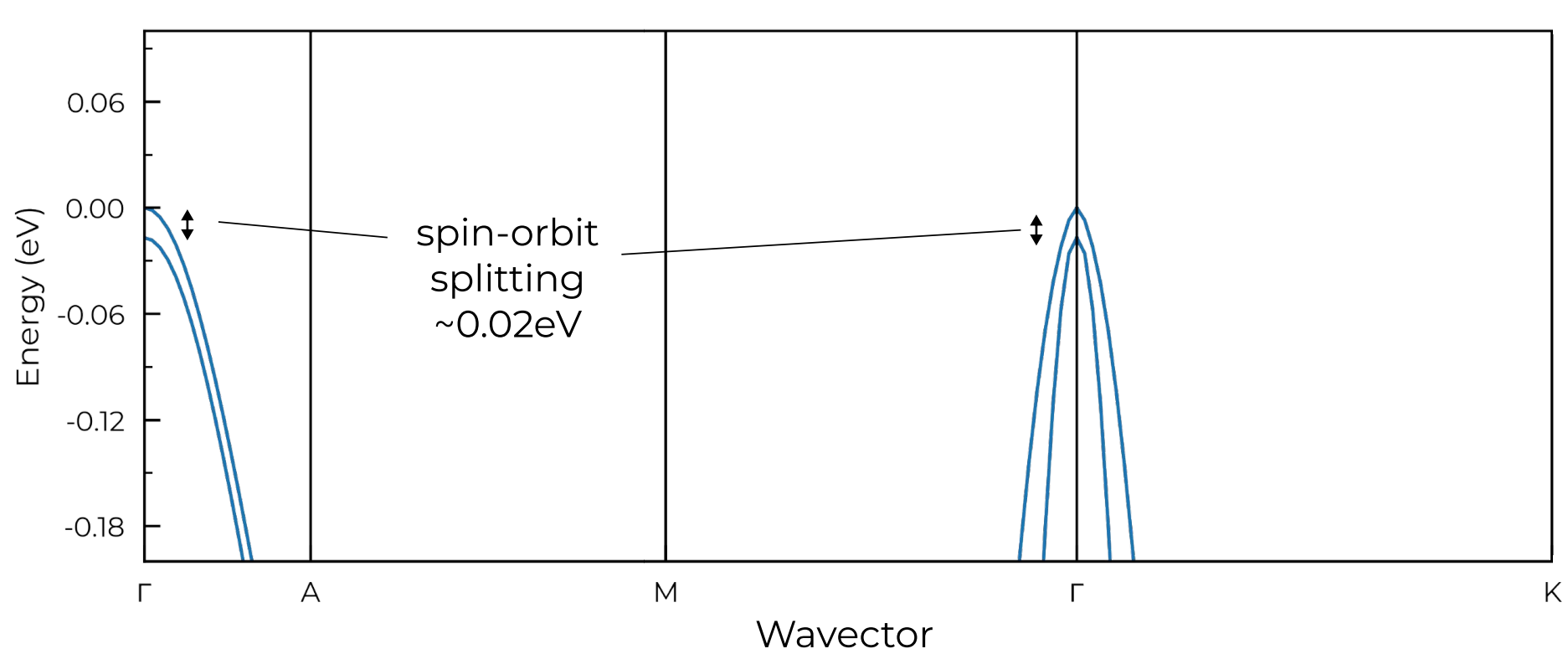

FIG. 5. Top of the valence band showing the splitting of the valence band minimum with the inclusion of spin-orbit coupling (SOC).

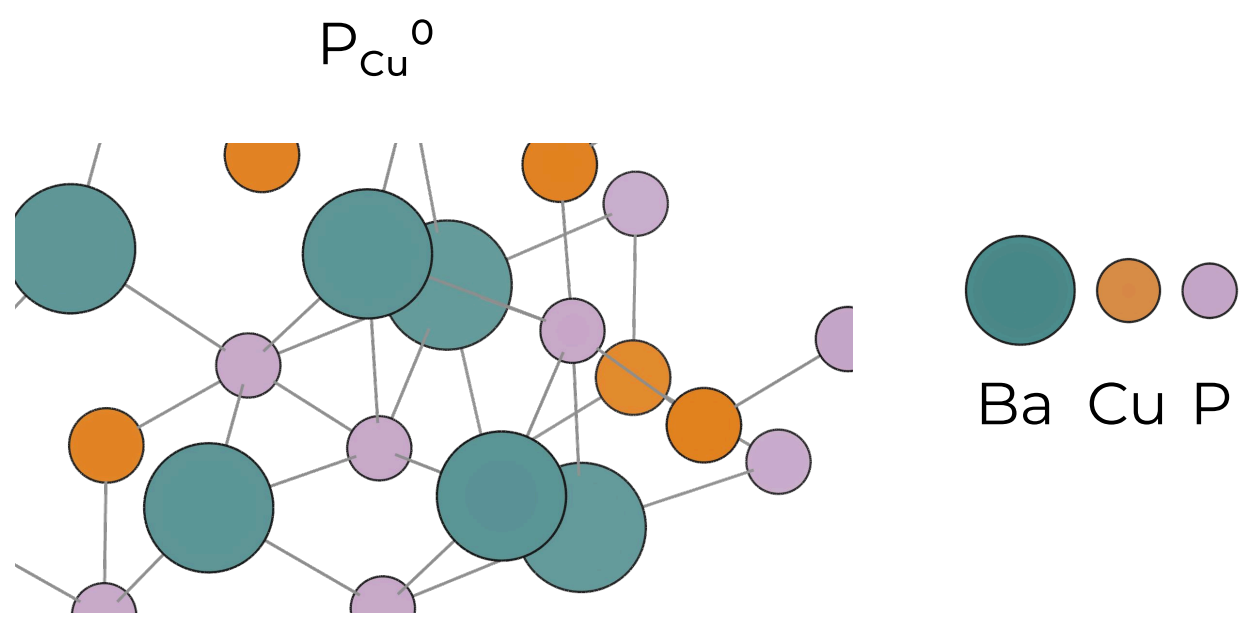

FIG. 6. Relaxed antisite P_{Cu} (0 charge state) showing the P-P dimer bond.

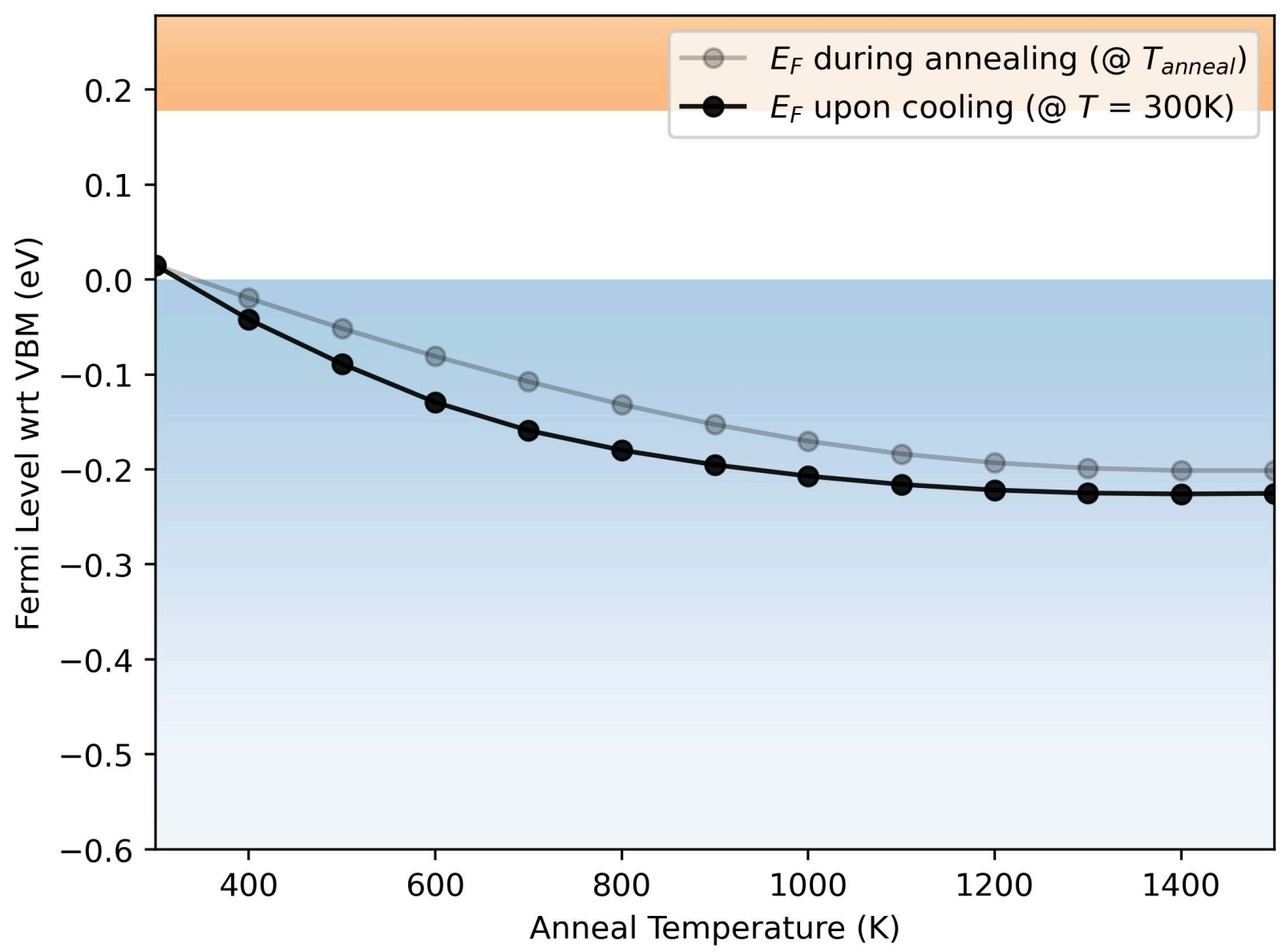

FIG. 7. Calculated self-consistent Fermi level positions in BaCuP during annealing (gray) and upon cooling to 300K (black). The light blue region represent the valence bands and the orange region the conduction bands.

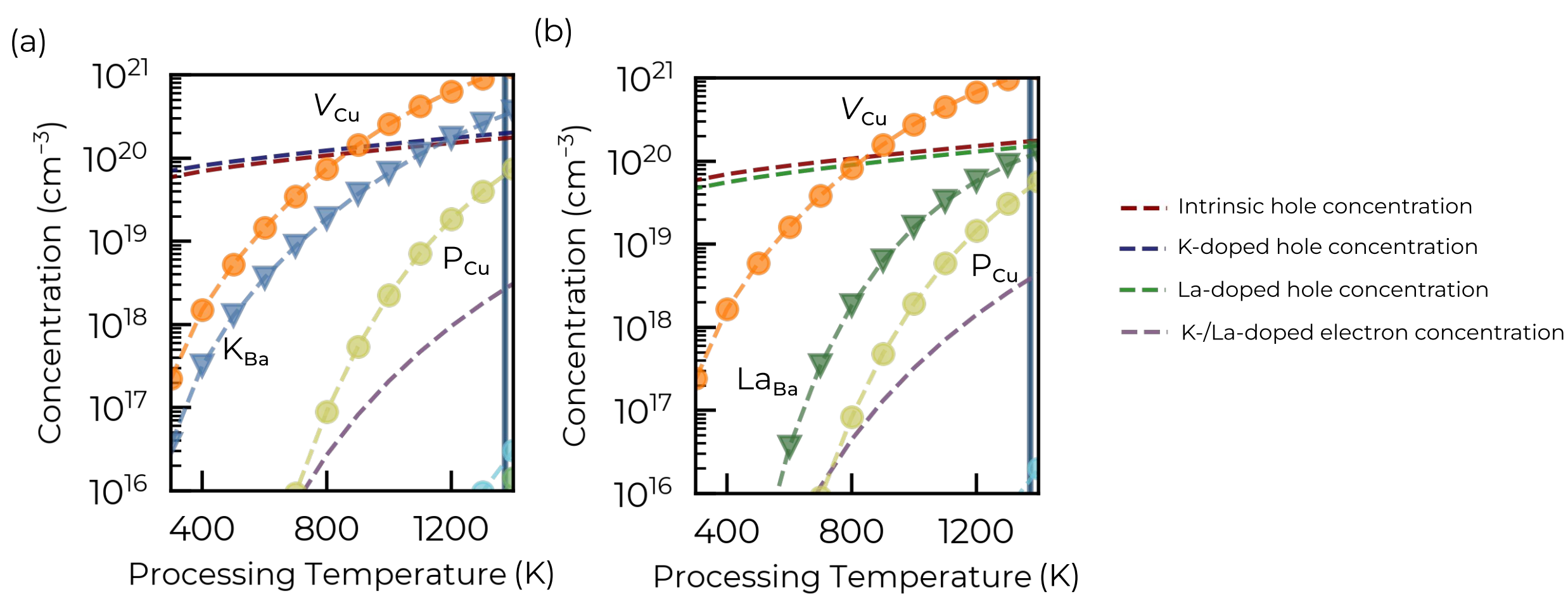

FIG. 8. Carrier and defect concentrations of (a) K-doped and (b) La-doped BaCuP, showing the relatively low electron concentration at operating temperatures.

Hole mobility ($cm^2\,V^{-1}\,s^{-1}$)			
	no SOC	SOC	Δ (%)
μ_{xx}	182.83	183.47	+0.4
μ_{yy}	182.83	183.47	+0.4
μ_{zz}	40.65	39.75	−2.2
μ_{avg}	135.44	135.56	+0.1

TABLE I. Effect of spin–orbit coupling (SOC) on the hole mobility of BaCuP at 10^{15}cm^{-3} and T =300 K. Mobilities are reported along the principal crystallographic directions and as the directional average, $\bar{\mu} = (\mu_{xx} + \mu_{yy} + \mu_{zz})/3$. AMSET interpolation grids were converged independently for each calculation ($119 \times 119 \times 49$ without SOC, $63 \times 63 \times 25$ with SOC, both starting from a $12 \times 12 \times 6$ **k**-point grid). The relative deviation is given in the last column.

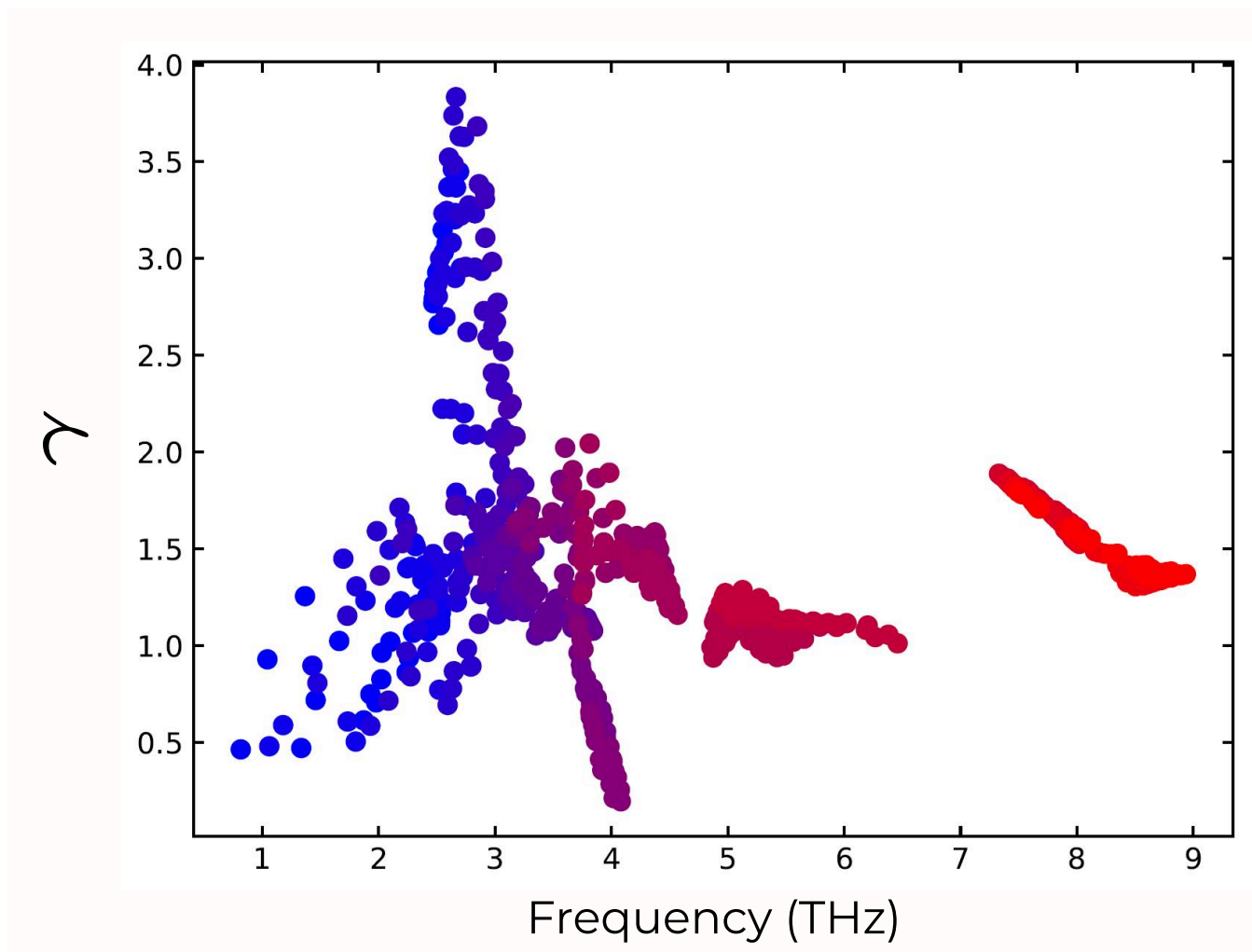

FIG. 9. Grüneisen parameter with respect to the frequencies.

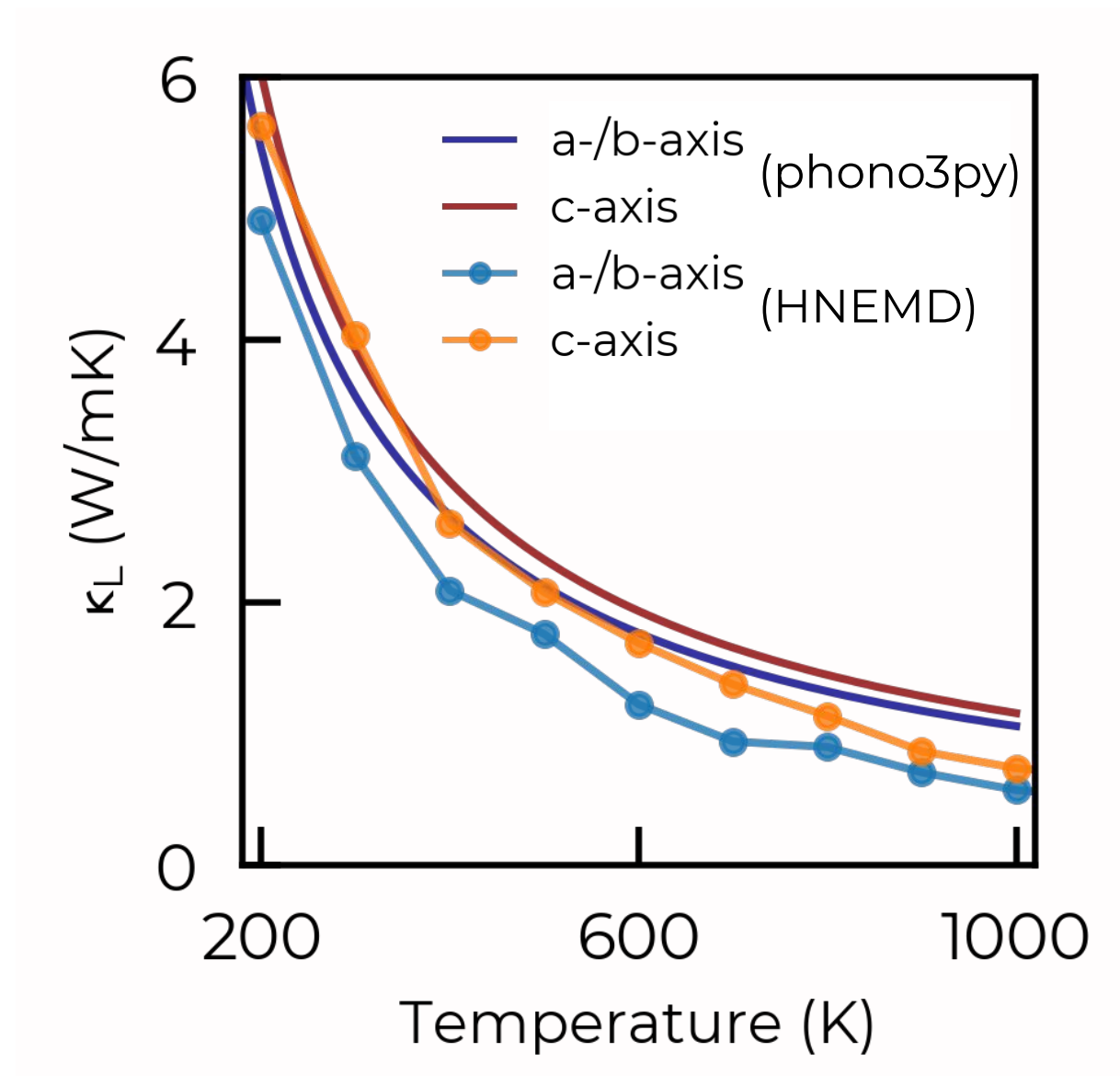

FIG. 10. Comparison of the lattice thermal conductivity obtained with phono3py and the HNEMD method.

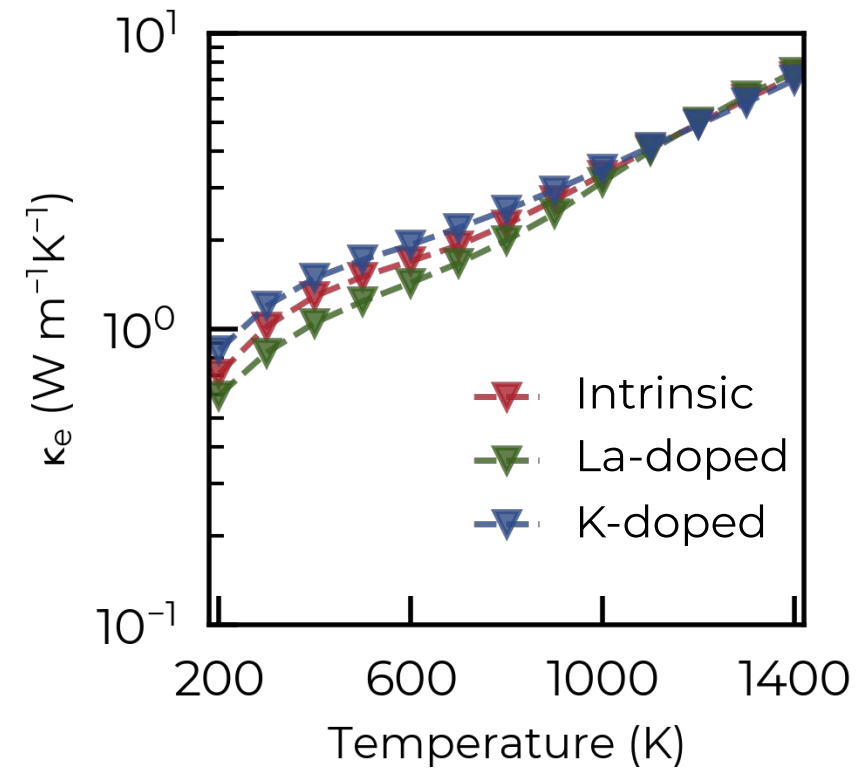

FIG. 11. Electronic part of the thermal conductivity computed with AMSET for BaCuP, La-doped and K-doped BaCuP.

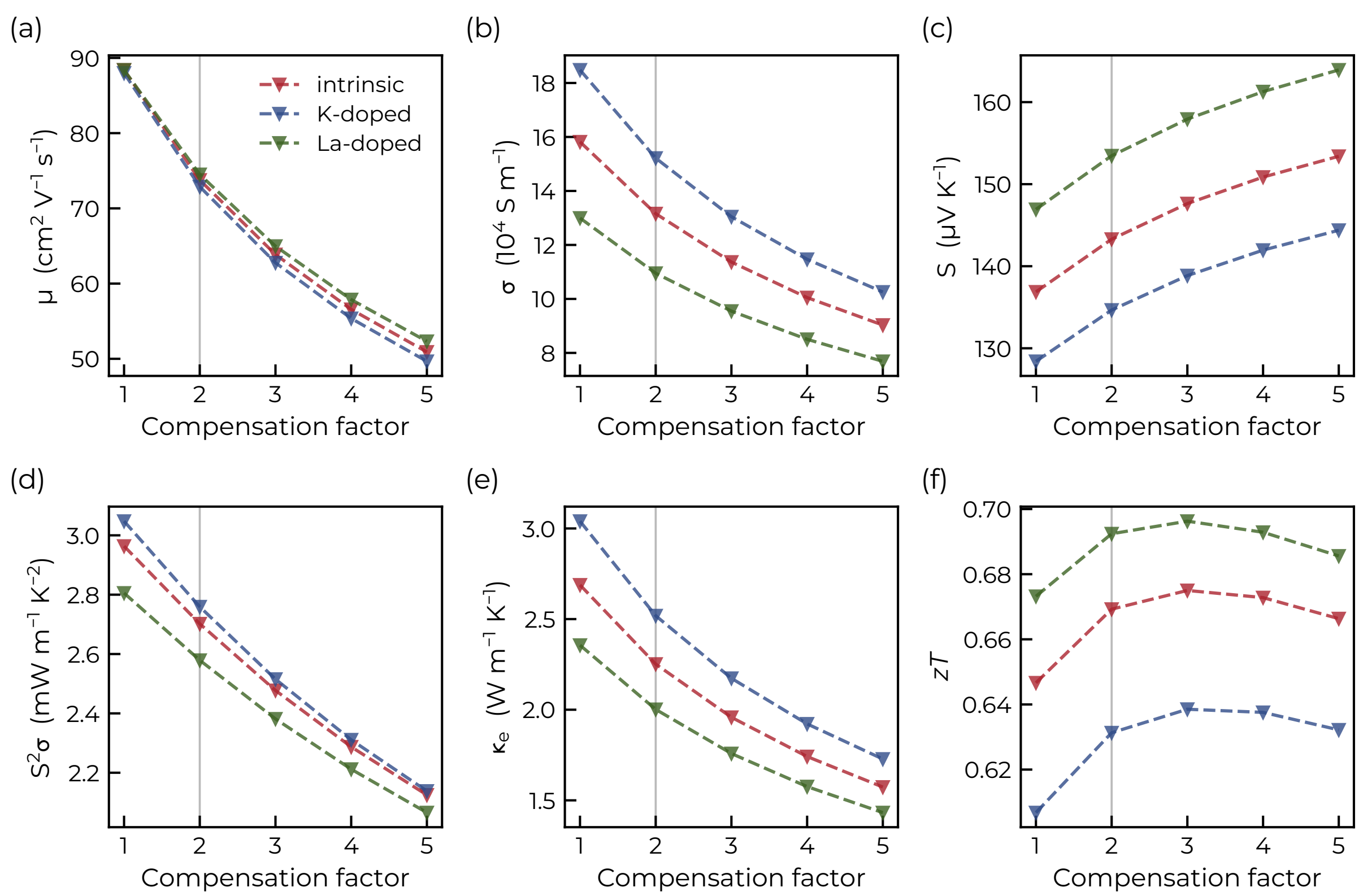

FIG. 12. Effect of the ionised-impurity compensation factor C on (a) hole mobility, (b) electrical conductivity, (c) Seebeck coefficient, (d) power factor, (e) electronic thermal conductivity and (f) figure of merit of intrinsic, K-doped and La-doped BaCuP at 800 K. The vertical line marks the value used throughout this work.

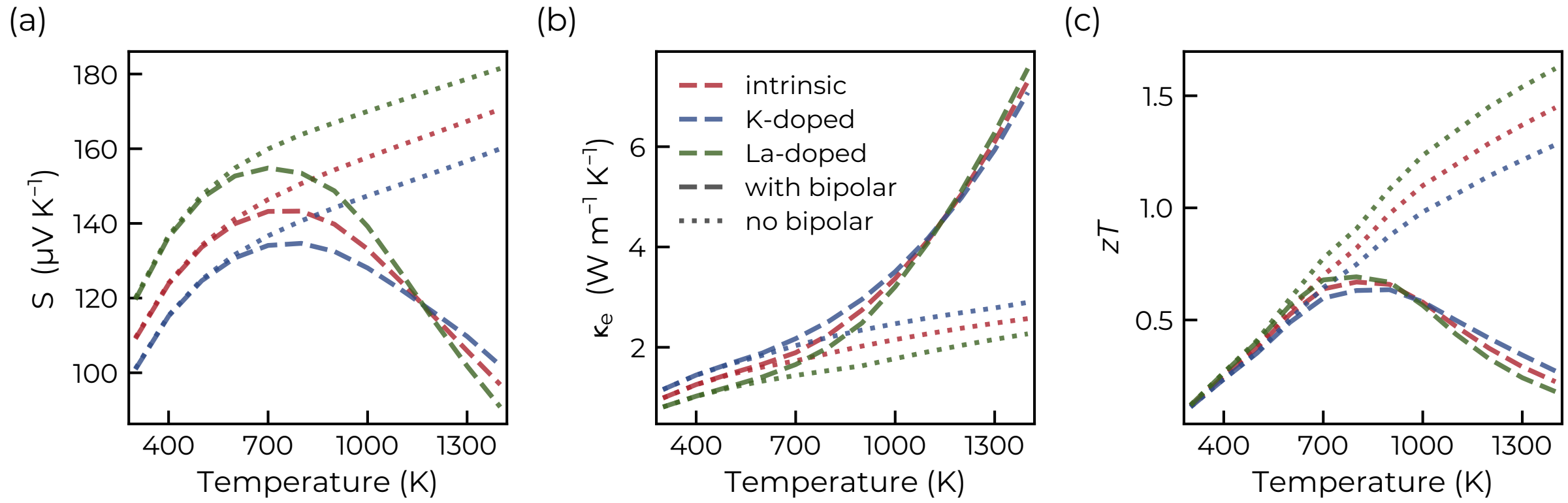

FIG. 13. (a) Seebeck coefficient, (b) electronic thermal conductivity and (c) figure of merit zT against temperature, computed with the calculated band gap (dashed, "with bipolar") and with the gap opened to 2 eV using a scissor operator (dotted, "no bipolar"), which suppresses most of the thermally excited minority carriers.

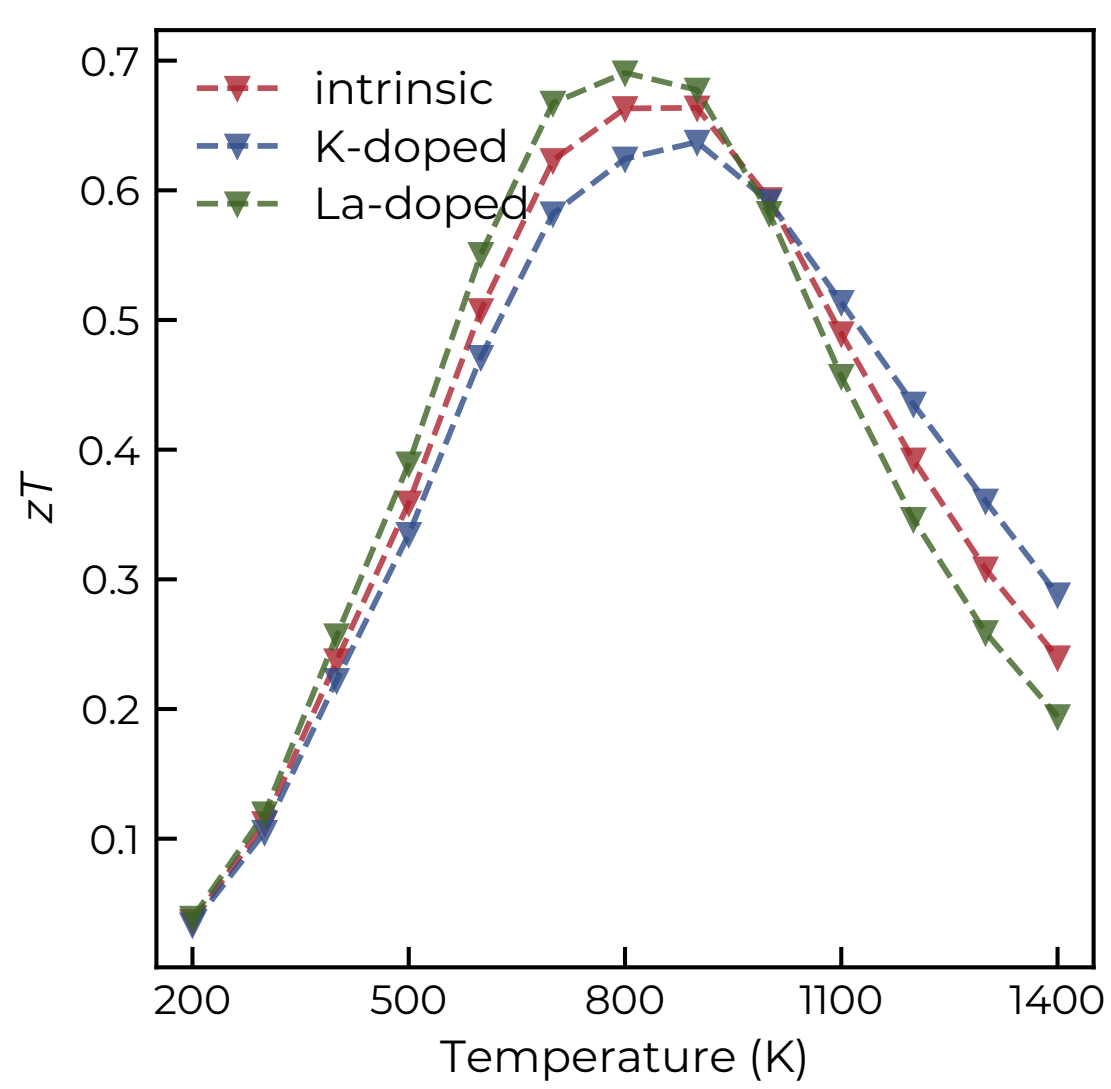

FIG. 14. Directionally averaged figure of merit zT against temperature for intrinsic, K-doped and La-doped BaCuP.

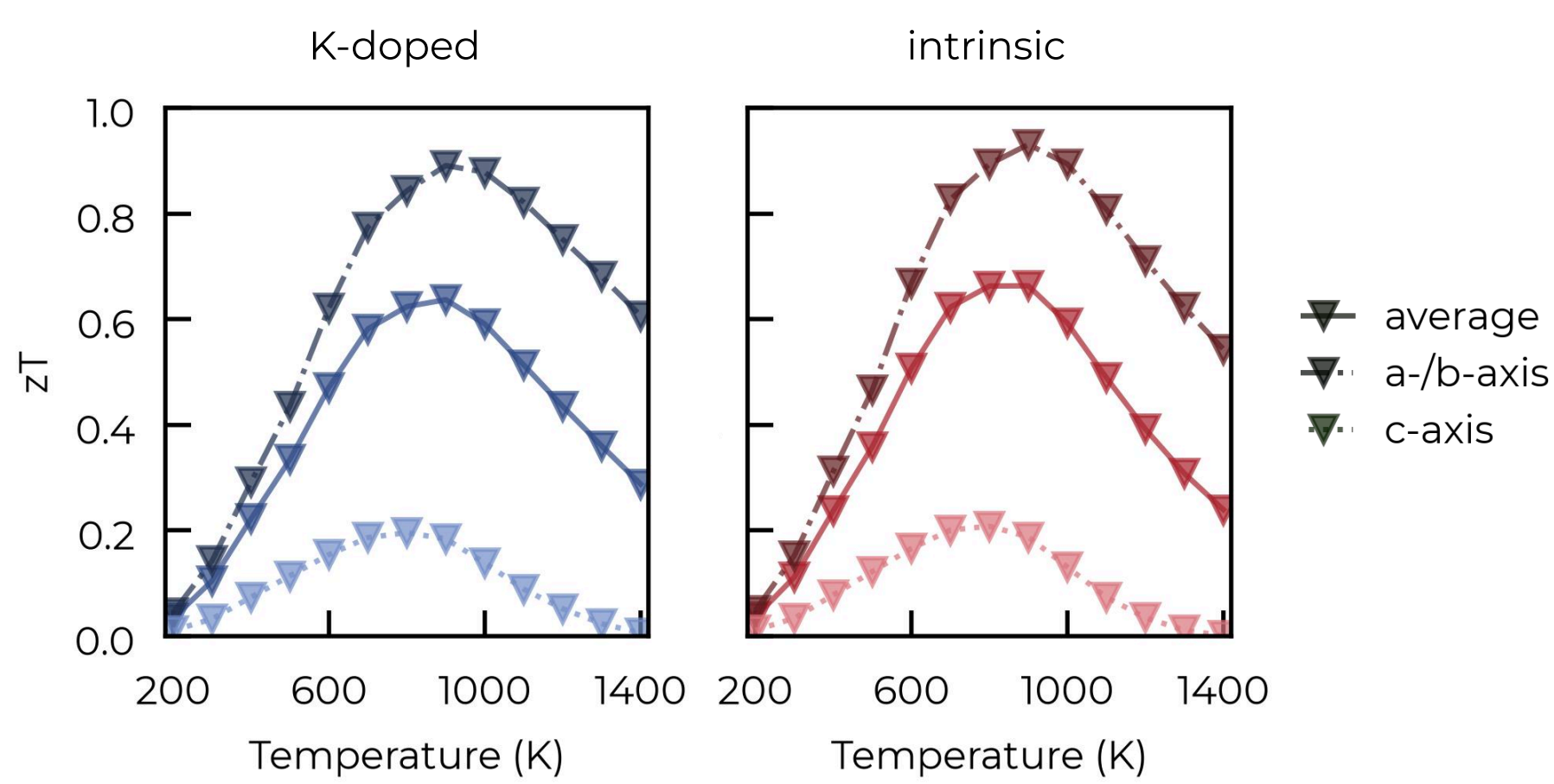

FIG. 15. Average, in-plane (a-/b-axis) and out-of-plane (c-axis) figure of merit zT against temperature for K-doped and intrinsic BaCuP. The equivalent data for La-doped BaCuP are shown in Figure 6(b) in the main text.

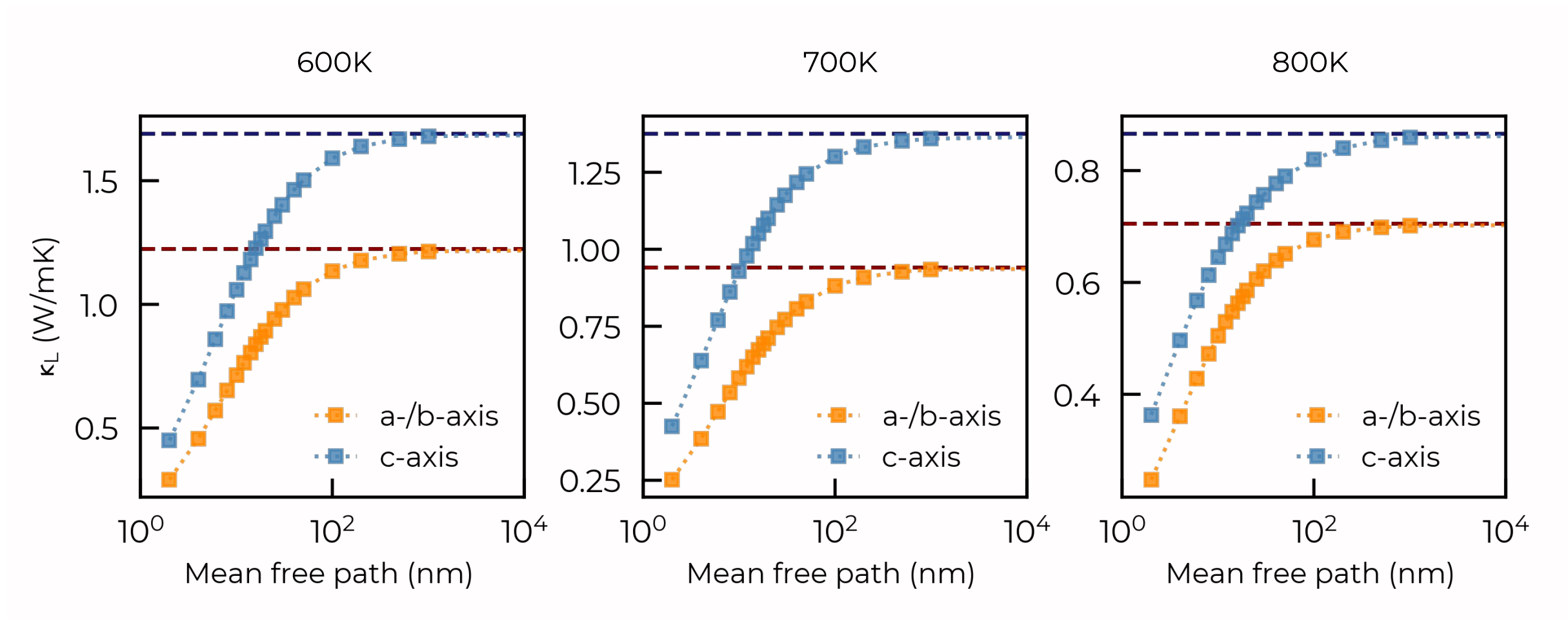

FIG. 16. Lattice thermal conductivity against the mean free path in the in-plane (*a*-/*b*-axis) and out-of-plane (*c*-axis) directions for different operating temperatures. The dashed lines correspond to the bulk values.

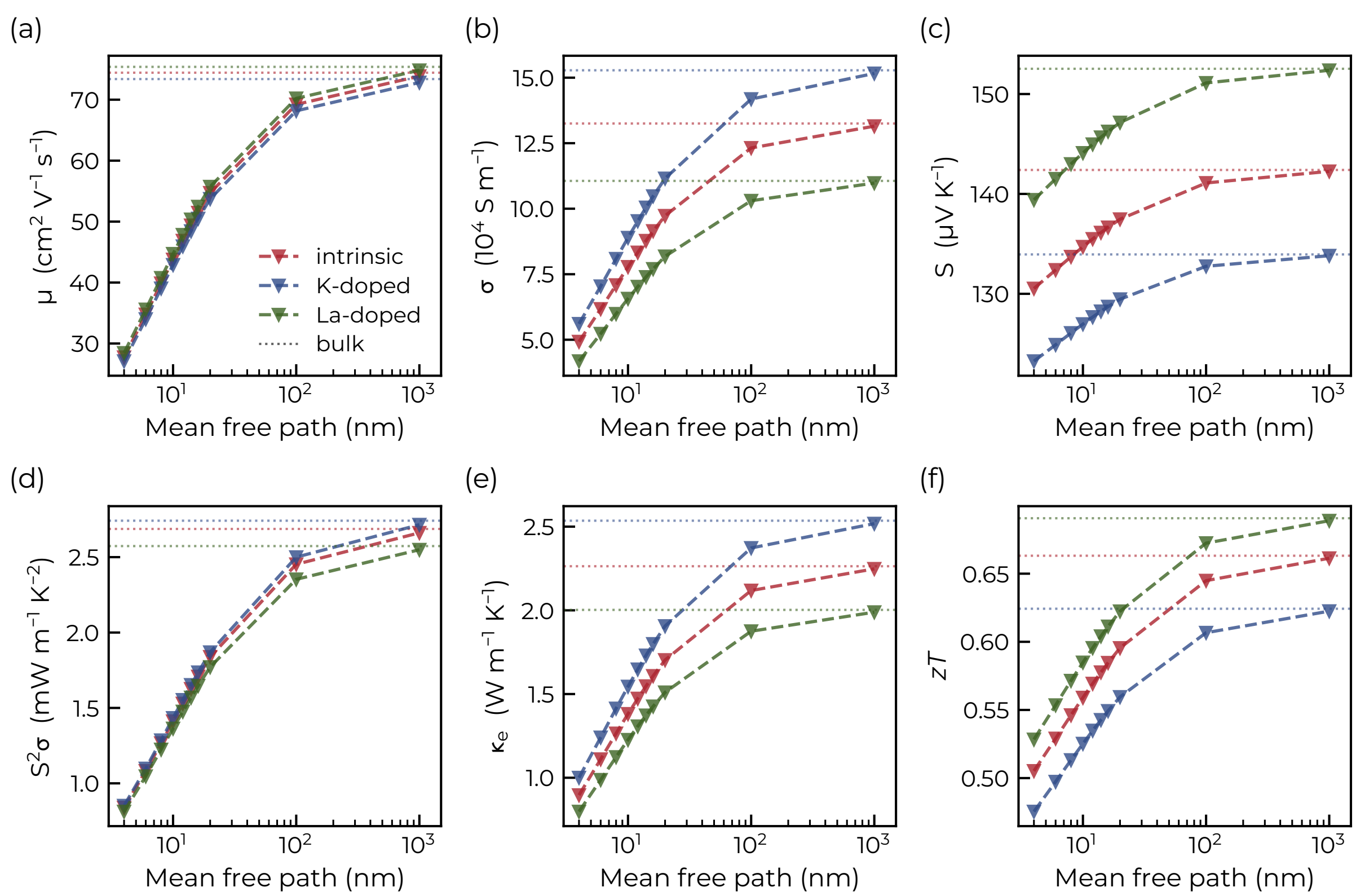

FIG. 17. (a) Hole mobility, (b) electrical conductivity, (c) Seebeck coefficient, (d) power factor, (e) electronic thermal conductivity and (f) figure of merit against the mean free path (in nm) at 800 K. Dotted horizontal lines give the bulk values.
